# Supplementary material for: Model-based analysis of sample index hopping reveals its widespread artifacts in multiplexed single-cell RNA-sequencing
Source: Nat Commun. 2020 Jun 1;11:2704. doi: 10.1038/s41467-020-16522-z (PMC7264361; doi:10.1038/s41467-020-16522-z)
Supplement: Supplementary file 1 — Supplementary Information [file 41467_2020_16522_MOESM1_ESM.pdf]

Supplementary Information

Model-based analysis of sample index hopping reveals its widespread artifacts in multiplexed single-cell RNA-sequencing

Farouni et al.

Supplementary Note 1

Overview of Index Hopping

Patterned flowcells. Ever since the emergence of next-generation sequencing technologies in 2006, the speed and throughput at which we can perform whole-genome DNA and RNA sequencing have been steadily increasing. For example, in recent years Illumina has introduced a family of sequencing platforms (i.e. the HiSeq 4000 and NovaSeq 6000) characterized by substantial increased capacity, faster run times, and lower sequencing cost. The significant boost in sequencing throughput[1] first came about in 2015 when Illumina introduced the patterned flow cell technology on the HiSeq 4000 sequencers. In particular, there were two key innovations that led to the significantly improved performance. (1) A flow cell surface design that optimizes the prearranged spacing of nanowells, thus allowing the accurate imaging of billions of clusters of amplified cDNA fragments. (2) *The Exclusion Amplification* (ExAmp) chemistry that instantaneously amplifies a single cDNA fragment, effectively excluding and preventing other cDNA fragments from forming a cluster within the same nanowell. In 2017, Illumina launched the NovaSeq 6000 instrument with an updated flow cell design that further decreased the spacing between the nanowells, thus significantly increasing the density of generated clusters and, consequently, the amount of data that can be generated. Currently, a total of 2.5B single reads can be generated on each of the four lanes that comprise a single NovaSeq S4 flow cell, compared to 350M reads on each of eights lanes of HiSeq 4000.

Sample multiplex sequencing. However, given that for most sequencing studies, the number of reads required to achieve an adequate transcriptome coverage tends to be in the tens of millions, it then becomes necessary to resort to *sample multiplex sequencing* strategies to fully utilize the massive capacity and cost efficiency that the patterned flow cell with ExAmp chemistry technology can bring about. The process of sample multiplexing can be briefly summarized with the following few steps. First, a *sample barcode index* is added to each cDNA fragment during library preparation (to one end in *single-indexing* or to both ends in *dual-indexing*). Then, cDNA fragments from multiple samples are subsequently pooled together in the same lane to be cluster-amplified and sequenced. Finally, the generated sequenced reads are demultiplexed into their respective source samples using the sample barcode indices. Note that with current technology, in a single run of droplet-based RNA-seq experiment, a maximum of a 384 samples can be multiplexed (Chromium i7 Multiplex Kit) using a 96-plex on each of the four lanes of a NovaSeq S4 flow cell. In what follows, we define a *sequencing library* as a collection of index-barcoded and PCR-amplified cDNA fragments which are purified from the mRNA of a particular sample tissue

Sample read misassignment. Unfortunately, sample multiplexing can cause a significant percentage of the demultiplexed sequenced reads to be misassigned to an incorrect sample barcode. Although sample read misassignments can arise due to several factors [1], one specific mechanism termed *sample index hopping* is the primary cause of read misassignments in patterned flow cells. Index hopping is believed to result from the presence of free-floating

[1] As measured by the total number of nucleotide sequence reads generated in a single run of an experiment

indexing primers that attach to the pooled cDNA fragments just before the exclusion amplification step that generates clusters on the flow cell. In addition, if during library preparation, free adapters or primers are not properly removed, the resulting purified library would show higher levels of index hopping that increases linearly with the molar concentration of free adaptors relative to DNA input [2].

Discovery and Quantification of Index Hopping. The problem of index hopping was first identified as early as December 2016 in a blog post reporting read sample misassignment on HiSeq 4000 and HiSeqX platforms [3]. Soon afterwards, Illumina released a white paper [2] acknowledging that index switching does indeed occur and tends to be higher in machines that use a patterned flow cell, but maintaining that the phenomenon affects only <2 % of reads. However, around the same time, a study [4] reported that 5-10% of sequencing reads were incorrectly assigned a sample index in a multiplexed pool of plate-based scRNA-seq samples. More recently, using two plate-based scRNA-seq datasets, a lower estimate of the index swapping on the HiSeq 4000 at approximately 2.5% was determined [5]. Another study [6] conducted in the context of exome sequencing also reported a contamination (index hopping rate) ranging from 2% to 8%. Using unique antigen receptor expression, [7] estimated the index hopping rate in plate-based single cell RNA-seq data (i.e. spread-of-signal across wells) to be approximately 3.9%. They also failed to detect any evidence that sample barcode indices vary in their proneness to undergo index swapping than others. With the aim of reconciling the conflicting hopping rate estimates that had been reported, a comprehensive study [8] used a non-redundant dual indexing adapters developed in-house and performed an exhaustive study across multiple libraries (whole genomes, exome, and stranded RNA) and sequencer models (HiSeq 4000, NovaSeq 6000 and HiSeqX) to determine the rate of index hopping. They observed a rate of 0.2% to 6% in all sequencing runs. More importantly, they showed that even in bulk RNA-seq libraries where the indexing hopping rate was as low as 0.32%, spurious results in downstream analysis can be generated. This finding mirrors the conclusions reached by the other five papers referenced in this paragraph including Illumina's white paper.

Supplementary Note 2

Mitigation Strategies

Experimental Strategies. Given that index hopping would occur in all sequencing platforms that uses patterned flow cells, one can resort to any of the following three experimental strategies to mitigate sample index hopping from affecting the integrity of experimental data.

1. *One sample per lane.* By avoiding multiplexing altogether and running one sample per lane, one can confine the sample indices in a given lane to one given sample only, thus minimizing the risk of index hopping from occurring in the first place. However, sample read misassignment can still occur due to other causes. Furthermore, this strategy can be financially prohibitive on high capacity platforms such as NovaSeq.

2. *Unique-at-both-ends dual indexing library.* By utilizing two unique barcodes for each sample, sample misassignment would occur only when both sample indices hop. Given the low probability that such an event would occur, index hopping can be reduced and computationally mitigated by discarding reads with unexpected combinations post-hoc. However, the strategy is currently incompatible with single index droplet-based protocols such as the widely used 10x Genomics single cell protocol.

3. *Post-library prep treatment.* By using Illumina's Free Adapter Blocking Reagent, the 3′ ends of the free adapters become blocked preventing their extension and thus reducing the rate of index hopping[2]. Nonetheless, the blocking reagent Illumina provides is only compatible with a few bulk DNA and RNA protocols, but not for single cell protocols. Whether it can be incorporated in single cell droplet-based protocol is yet to be determined.

Computational Strategies. Given the limitations of existing experimental strategies to eliminate index hopping, the urgency to develop computational strategies instead has only intensified since the phenomenon was first discovered. Indeed, the first attempt to computationally correct for index hopping in multiplexed sequencing libraries was only recently published[9]. However, the linear regression method the authors propose is applicable only to plate-based scRNA-seq samples, where a unique combination of a pair of sample barcodes in each well determines the identity of cells and can give rise to crosshair pattern in the data when index hopping occurs. It is this pattern that allows the estimation of fraction of hopped reads and upon which their proposed approach relies. Another limitation of their approach is that it uses only a subset of the data corresponding to genes whose specific cell expression is above a given threshold. However, a more accurate method for plate-based scRNA-seq that makes fewer assumptions and that uses data from all the genes was developed[5]. In that same paper, the authors also proposed a heuristic computational strategy for droplet-based scRNA-seq 10x Genomics experiments that excludes hopped reads without removing the corresponding cell libraries, but did not attempt to propose a modeling framework for estimating sample index hopping in droplet-based data since unlike plate-based assays, droplet-based assays do not use a unique pair of sample barcodes but rather use one mate of the paired read for quantification and a second mate to carry the UMI and cell barcode tags, thus rendering the crosshair pattern approach inapplicable.

Supplementary Methods

Method's Scope. Although much of what we propose in this paper can be ported, modified, and applied to other protocols, we limit the scope of the proposed approach to droplet-based scRNA-seq data, with libraries prepared using the 10x Genomics Single Cell Protocol and that are subsequently multiplexed for sequencing on Illumina machines.

Section Outline. The first four subsections of the **Supplementary Methods** correspond to the following goals: (1) building a generative model that probabilistically describes the phenomenon of sample index hopping of multiplexed sample reads; (2) estimating the index hopping rate from empirical experimental data; (3) inferring the true sample of origin of hopped reads; (4) correcting for the effects of sample index hopping through a principled probabilistic procedure that discards predicted phantom molecules by optimizing the trade-off between false positive and false negative rates. The fifth and final subsection contains details of corresponding mathematical derivations.

3.1 Model Formulation

Sequencing Reads. Consider a droplet-based single-cell sequencing experiment where a total of S (ranging from 2 to 96) libraries are pooled together and multiplexed on the same lane of a patterned flow cell. In a single sequencing run, millions of short sequencing reads are generated (from 350M reads on a single lane of HiSeq 4000 to 2.5B reads on a single lane of a NovaSeq 6000 S4 flowcell), each of which is annotated with barcodes representing the sample, cell, and molecule from which the read originated. If the reads are aligned to the genome, a read would also be annotated with the genomic location where it mapped to. That is, after sample demultiplexing and transcriptome alignment, each read becomes associated with a *cell-umi-gene-sample* label. That is, each mapped read in a 10x Genomics Single Cell 3' v2 Gene Expression Library can be annotated by four labels: (1) *A sample barcode*, (2) *cell-barcode index*, (3) *Unique Molecular Identifier* (UMI) (4) *gene ID.*

1. *A sample barcode.* In the case of a 10x Genomics single-indexed library, there are four indices for each sample. In what follows, we collapse all four indices into a single sample index for computational, mathematical, and practical considerations. However, as we show in Section 3.5, the model we propose allows us to recover the sample index hopping rate at the level of the barcodes.

2. A 16bp *cell-barcode index* randomly selected out of a set containing 737,280 possible combinations. In scRNA-seq data, a cell is identified by a unique cell barcode. However, only a fraction (1k-10k) out of the +100K observed cell barcodes correspond to actual cells. In each of these cells, we typically observe anywhere from a minimum of a 1000 (a threshold usually specified manually) up to approximately 100,000 unique molecules, or more depending on the average read coverage per cell.

3. A 10bp *Unique Molecular Identifier* (UMI) index for which there are a total of $4^{10} = 1,048,576$ combinations. A *UMI collision* occurs when two or more UMIs possess the same sequence. Note that given that the UMI index in v2 chemistry is 10bp long, the number of possible UMIs is one order of magnitude larger than the number of UMIs typically observed in any one cell. Accordingly, a UMI collision in a single cell, when it does occur, would result in two reads from different molecules to be considered as originating from the same cDNA fragment (i.e. PCR duplicates), thus reducing the number of real molecules that can be observed in any one cell. To further reduce the chance of collisions, gene ID labels can be considered in order to

limit the space of collisions to those UMIs that are mapped to the same region of the genome. Although these genomic regions, each consisting of the union of exons belonging to a given gene, vary in length, the localization of possible collisions to a small region of the genome greatly reduces the probability of UMI collisions.

4. A mapped *gene ID* as provided by a transcriptome. For example, Ensemble 95[10] has annotations for 19,768 and 21,823 non-conjoined coding and noncoding genes, respectively. We note here that by discarding unmapped reads and reads that mapped to genomic regions other than exon bodies, we are further making the implicit assumptions that the large portion of data that we end up retaining contains enough information to determine the index hopping rate and that the data we discard is not characterized by a different mechanism underlying the sample barcode index hopping phenomenon.

Modeling Sequencing Reads. We start by observing that after cDNA molecules of a given transcript are amplified, they are subsequently fragmented into 300-400 bp fragments, each containing both a cell barcode and a UMI. During the sample index PCR amplification step, an Illumina Read 2 adapter is ligated to a fragment m before it gets amplified, resulting in n_m sequencing reads having the same *cell-umi-gene-sample* label. If none of the n_m sequencing reads are misassigned, then each n'th sequencing read's observed sample index d_{mn} would correspond to the true sample index s_{mn}. When index hopping occurs, the read misassignment process can be modeled as a mixture of S categorical distributions where each observed molecule's read sample index belongs to a given sample. See **Supplementary Table 9** for an example toy data table in which each row corresponds to a single sequencing read with label m.

We can formulate the mixture model formally as a two-stage hierarchical sampling process, where we first draw a molecule with an unobserved true sample index s_{mn} that gets assigned to read n with label m from a categorical distribution governed by a label-specific probability parameter vector $\boldsymbol{\varphi}_m$. Next we draw the observed sample index from another categorical distribution with a probability parameter vector $\boldsymbol{p}_{s_{mn}}$ whose value is conditional on the value of s_{mn}. In what follows, s_{mn} denotes the index of the element in the vector $\boldsymbol{s_{mn}}$ for which we observe a one (i.e $[\boldsymbol{s_{mn}} = 1]$).

$$
\begin{aligned}
\boldsymbol{s}_{mn} &\sim \text{Categorical}(\boldsymbol{\varphi}_m) \\
\boldsymbol{d}_{mn}|s_{mn} &\sim \text{Categorical}(\boldsymbol{p}_{s_{mn}}) \\
n = 1 \dots n_m&,\ m = 1 \dots M.
\end{aligned} \tag{5}
$$

Where $\boldsymbol{\varphi}_m \in [0,1]^S,\ \|\boldsymbol{\varphi}_m\|_1 = 1,\ \boldsymbol{p}_{s_{mn}} \in [0,1]^S, \left\|\boldsymbol{p}_{s_{mn}}\right\|_1 = 1,\ d_{mn} \in \{1, \cdots, S\},\ s_{mn} \in \{1, \cdots, S\},\ S \in \mathbb{N}_+$.

The sample hopping probability matrix. The probability parameter vectors of all the S distributions can be stacked together in $\boldsymbol{P}$ as such.

$$
\boldsymbol{P} = \begin{bmatrix} \boldsymbol{p}_1^\intercal \\ \boldsymbol{p}_2^\intercal \\ \vdots \\ \boldsymbol{p}_S \end{bmatrix} = \begin{bmatrix} p_{11} & p_{12} & \cdots & p_{1S} \\ p_{21} & p_{22} & \cdots & p_{2S} \\ \vdots & \vdots & \ddots & \vdots \\ p_{S1} & p_{S2} & \cdots & p_{SS} \end{bmatrix}
$$

When $\boldsymbol{P} = \mathbf{I}$, then the probability that a read keeps its sample index of origin is one, and consequently, reads do not hop over to other samples. That is, $d_{mn} = s_{mn}$ for all m and n. Here p_{ij}

denotes the probability that a read from sample i hops to sample j. The number of parameters in $\boldsymbol{P}$ equals $S \times (S-1)$. Furthermore, in Model 5 there are a total of M parameter vectors $\boldsymbol{\varphi}_m$ each taking values on the probability simplex for a total of $M \times (S-1)$ parameters. We can reduce the number of parameters from $(M+S) \times (S-1)$ to 1 by making the following two assumptions.

Toy Data Example To illustrate the concepts and definitions in the preceding sections, consider the following toy example where we have three samples $S = 3$. The data would thus consist of three-dimensional discrete vector observations $\boldsymbol{y}_l \in \mathbb{N}_0^3$ sampled from a multinomial distribution with total sum of counts r, where $\|\boldsymbol{y}_l\|_1 = r$, and a probability parameter.

$$\boldsymbol{p} \in \Delta^2 = \left\{ (p_1, \ldots, p_3) \in \mathbb{R}^3 \mid \sum_{s=1}^{3} p_s = 1 \text{ and } p_s \geq 0 \text{ for all } s \right\}$$

For concreteness, assume that the source sample s is known for each observation and consider what the data would look like when the sample index hopping rate (i.e. probability) is not zero (e.g. $\boldsymbol{p}_1 = [.9, .05, .05]$) such that the probability concentration bleeds into outcomes with hopped reads (see **Supplementary Table 10**). That is, we see that the same label we associate with a unique molecule co-occurs with more than one sample index. When there is no index hopping, for example, when **SIHR**$= 0$ (e.g. $\boldsymbol{p}_2 = [0, 1, 0]$), we observe reads only in one of the three samples (i.e. *non-chimeric non-fugues*). In **Supplementary Table 10**, we show observations corresponding to only 4 out of the 10 possible outcomes at read count level $r = 3$. However, when r is large, the set of possible outcomes increases exponentially. When $S = 3$, probabilities of observing these outcomes are given by the corresponding terms of the trinomial expansion of $\boldsymbol{p}$.

To better visualize the distribution of possible outcomes, consider the case at the read count level $r = 3$ when reads hop from one sample only, that is, when the true sample of origin is $s = 1$ (See **Supplementary Figure 8**). When $r = 3$ the number of coefficients and thus corresponding outcomes (i.e. elementary events) are given by the $(r+1)$'th *triangular number* $t = \binom{r+(S-1)}{(S-1)}$, which here equals $\frac{(3+1)\times(3+2)}{2} = 10$. That is, we group the 10 outcomes, which make up the sample space $\mathcal{Y}_r$ into a set of three categories: (1) Three non-chimeric outcomes, each lying on one of the three $0-$faces (vertices); (2) Six $2-$chimeric outcomes, two lying on each of the three 1-faces (edges); (3) One $3-$chimeric outcome lying in the middle of a single $2-$face (facet) as shown in Figure 1. In general, the number of $m-$faces of an $r-$simplex is $u = \binom{r+1}{m+1}$. The unit probability measure is split over r outcomes instead of t outcomes. Out of the u $m-$faces, $t = \binom{r}{m}$ correspond to non-fugue outcomes whereas $f = (u - t)$ are fugues (i.e. those observations where all the reads hop from their source sample). That is, if in **Supplementary Figure 8**, the true sample of origin is $s = 1$, then a 1-read hop would correspond to two possible outcomes: two 2-chimeric observations each giving rise to one true and one phantom molecule; A 2-read hop would correspond to three possible outcomes: two 2-chimeric observations each giving rise to one true and one phantom molecule and a single 3-chimeric observation giving rise to one true and two phantom molecules; A 3-read hop would would correspond to four possible outcomes: two 2-chimeric fugue observations each giving rise to two phantom molecules and a two non-chimeric fugue observations, each giving rise to one phantom molecule. Note however that when we consider hopping from all S samples, the number of possible outcomes e increases by a factor of S, so when $r = 3$, we have $e = 30$ possible outcomes.

The number of observations in data $\mathcal{Y}$ is typically high (in the hundreds of millions). Each observed vector of read counts can be categorized as a combination of *chimeric/non-chimeric* and *fugue/non-fugue*. For each k-chimeric observation, we potentially have from one and up to $S - 1$ *phantom molecules*. For example, the observation $\boldsymbol{y}_4$ has two *phantom molecule* and zero real

molecules. If there is indeed no index hopping, then the number of *phantom molecules* would be zero whereas the number of real molecules would equal to L, the number of unique label combinations. Although the probability that all reads get misassigned to the same sample is negligibly low, the effect can be significant for non-chimeric labels for which there is only one or two reads (a group that makes up a large fraction of observations).

Two Simplifying Assumptions. The n_m reads associated with label m can come from different source samples. This could happen for example when reads from two samples are assigned the same label simply by chance. However, such an outcome is extremely unlikely and by ruling it out we can greatly simplify the modeling framework if we constrain each of all the M parameter vectors $\boldsymbol{\varphi}_m$ to be the one vector (i.e. $\mathbf{1}$). More formally, we formulate the assumption as follows.

Assumption 1 (Discrete Simplex Constraint Assumption)**.** Let $max(\boldsymbol{\varphi}_m) = 1$ for all l such that $\boldsymbol{\varphi}_m \in \Delta_1^S := \left\{ \left(\ \varphi_0, \ldots, \varphi_S \ \right) \mid \varphi_i \in \{0,1\}, \sum_i \varphi_i = 1 \right\}$. That is, we restrict $\boldsymbol{\varphi}_m$ to belong to the discrete S-simplex, a set with cardinality S whose elements are vectors each consisting of a single 1 and zeros elsewhere. We basically assume that all the reads with a given *cell-umi-gene* (CUG) label (i.e. PCR read duplicates of a unique cDNA molecule) can be indexed with the same sample barcode only.

As a result of Assumption 1, the sample index s_{mn} becomes a constant random variable drawn from one of S degenerate categorical distributions such that all the n_m reads with label m are assigned the same sample index. That is, for each $\boldsymbol{\varphi}_m$, all the $\boldsymbol{p}_{s_{mn}}$ vectors become the same for all the n_m reads (i.e. $\boldsymbol{p}_{s_m}$). Assumption 1 entails the following two corollaries.

Corollary 1 (Label Collision Across Samples)**.** A given *cell-umi-gene* label combination has a zero probability of co-occurring with more than one sample barcode index. That is, reads annotated with the same label combination can only belong to one sample.

Corollary 2 (Number of Molecules)**.** The number of total molecules across all S samples equals the number of unique *cell-umi-gene* label combinations L. That is, each CUG would represent one unique molecule and all sequencing reads with the same CUG would correspond to PCR amplification products of that original molecule. In other words, the number of molecules equals the number of rows in a merged data table of sample read counts, which has been fully joined by the combination of cell, umi, and gene key (**Supplementary Notes**).

Furthermore, given that we have no reason to believe that reads in a particular sample are characterized by a different chemical properties, we can simplify the problem by making a second assumption.

Assumption 2 (One parameter to rule them all)**.** The probability of a read keeping its sample index is the same across samples (i.e. p) and the probability of a read switching the sample index is the same regardless of either its source or target sample (i.e. $p_h = \frac{(1-p)}{S-1}$).

Assumption 2 reduces the number of parameters in P from a total of $Q = (S-1) \times S$ to just a single parameter.

$$\boldsymbol{P} = \begin{bmatrix} p & p_h & \cdots & p_h \\ \vdots & \vdots & \ddots & \vdots \\ p_h & p_h & \cdots & p \end{bmatrix}$$

More importantly, Assumption 2 induces a symmetry on the probabilities we assign to the possible counts that lie within the same $m-$face of an $(S-1)-$simplex denoted by Δ_r^{S-1}, which in turn constitutes the r'th component of the Pascal's Simplex denoted by $\wedge^S$ (See **Methods**).

The Molecular Proportions Complexity Profile. In a multiplexed experiment, several samples that vary in their library complexity are sequenced together, where we define *library complexity* as the expected number of unique molecules sampled with a finite number of sequencing reads generated in a given high-throughput sequencing run. These samples would differ in the total number of unique transcripts each one has due to a host of factors, ranging from the presence of varying amounts of RNA that characterize different cell types (e.g. neuronal cells have low RNA content) to accidental errors in library preparation that could cause many cells to break up and lose their endogenous mRNA. That is, even if total number of available sequencing reads were budgeted evenly over the multiplexed samples, the number of unique molecules detected in the sequencing run could vary widely across the samples. In order to assess and identify potential problems such as low library complexity across all the samples in a sequencing run, we propose the that a more informative picture of the library quality of samples could be gained, if we consider the molecule counts conditional on the *PCR duplication level* r or more specifically, the set of molecular proportions $\Pi := \{\boldsymbol{\pi}_r\}_{r=1}^{R}$, which we term the *molecular proportions complexity profile*. We can obtain an estimate for π_{rs} from the observed proportion of read counts $\bar{v}_{rs}$ for sample s observations at *PCR duplication level* r from Equation 10 in **Supplementary Methods 2**. These estimates are used as plug-in parameters in Model 1 in **Methods**.

The molecular proportions complexity profile plots for four datasets we analyzed are shown in **Supplementary Figure 5** . The same three samples (B1, B2, and D2) in both the HiSeq 2500 and HiSeq 4000 datasets show molecular proportion curves indicative of low library complexity. Indeed even though the number of reads is approximately equal across the 8 samples, the number of molecules differ drastically, by an order of 100 or more, and are lowest in these three samples (see analyses notebooks). Furthermore, whereas the proportion curve of Sample D2 peaks at a moderate PCR duplicate level then tapers off, those of B1 and B2 start picking up and maintain a steady proportion for a wide range of high duplicate level values, up to $r = 1706$ and $r = 3744$, for HiSeq 2500 and HiSeq 4000, respectively. These trends are indicative of problematic samples; incidentally, Samples B1, B2 are the two samples that our analysis identifies to have the largest fraction of phantom molecules. As for the NovaSeq 6000 datasets, the curves for Samples $P7\_1$ and $P7\_8$ indicate low complexity libraries. This is also confirmed by the analysis since both samples turned out to have the two lowest number of molecules but the two highest fractions of phantom molecules among the 16 multiplexed samples.

Modeling Sequencing Reads Counts. Assumption 1 allows us to sum over the n_m categorical random variables to obtain a mixture of multinomials model for data $\{\boldsymbol{y}_m\}$, where $\boldsymbol{y}_m = (y_{m1}, \cdots, y_{ms}, \cdots, y_{mS})$ denotes the vector of read counts across S samples corresponding to an observed CUG label (i.e. molecule) $m = \{1, \cdots, M\}$ originating from an unobserved source sample $s_m \in \mathcal{S} := \{1, \cdots, S\}$. Here, the total number of CUGs (i.e. molecules) is $M \in \mathbb{N}_+$; the total number of PCR duplicated reads associated with molecule m is by $\|\boldsymbol{y}_m\|_1 = n_m$; and the total number of mapped reads across all samples in the dataset is $N = \|\boldsymbol{n} = (n_1, \cdots, n_M)\|_1$ where $\boldsymbol{n}$ is the M-dimensional vector of read count sums.

We are interested in specifying a model of read counts conditional on a given unique PCR duplication level. So in what follows, we partition $\boldsymbol{n}$ by the unique values that n_m can take. That is, the number of PCR duplicated reads - whether hopped or not - a given unique molecule generates, more specifically, $r \in \mathcal{R} := \{1, \ldots, R \mid r \in \mathbb{N}_+\}$, where $\|\mathcal{R}\| = I$ and R is the maximum value in $\mathcal{R}$. For each $r \in \mathcal{R}$, let $m_r \in \mathcal{M} := \{m_1, \ldots, m_R \mid m_r \in \mathbb{N}_+\}$ denote the corresponding number of times r is observed in $\boldsymbol{n}$ such that $\sum_{r=1}^{R} m_r = M$. We denote the empirical distribution of r by $\mathcal{M}(.)$. Accordingly, we can write the mixture of multinomials model conditional on the *PCR duplication level* r as follows.

$$
\begin{aligned}
\boldsymbol{s}_l &\sim \text{Categorical}(\boldsymbol{\pi}_r) \\
\boldsymbol{y}_l | \ \boldsymbol{s}_l &\sim \text{Multinomial}(r, \boldsymbol{p}_{s_l}) \\
l &= 1, \ldots, m_r.
\end{aligned} \tag{6}
$$

where l now indexes the m_r observations with *PCR duplication level* r; $\boldsymbol{y}_l \in \mathbb{N}_0^S$, $S \in \mathbb{N}_+$, $\|\boldsymbol{y}_l\|_1 = r$, $s_l \in s$, $\boldsymbol{\pi}_r \in [0,1]^S$, $\|\boldsymbol{\pi}_r\|_1 = 1$, $\boldsymbol{p}_s \in [0,1]^S$, $\|\boldsymbol{p}_s\|_1 = 1$; the vector $\boldsymbol{p}_{s_l}$ denotes the s_l row of the $S \times S$ sample hopping probability matrix P; and the probability vector $\boldsymbol{\pi}_r$ represents the proportion of molecules across the S samples at *PCR duplication level* r.

The mixture model can be viewed more intuitively as a generative process in which we first sample a molecule $\boldsymbol{s}_l$ from a library sample s_l according to the categorical model then we amplify the molecule by generating r PCR read duplicates according to the multinomial model. The number of molecules originating from a given sample with a given PCR read duplicates r is determined by the parameter vector π_r whereas the number of PCR duplicated reads that end up hopping to other samples is determined by the parameter vector $\boldsymbol{p}_{s_l}$).

Definitions. Corollary 2 implies that since a distinct molecule is defined by a read (or multiple reads) with a unique *cell-umi-gene* label, any label collision across samples would result in a total number of *observed* molecules greater than M. Therefore, to avoid any potential naming confusions, we make the following clarifying definitions (see **Methods** for concrete example).

Definition 1 (Chimeric Observation)**.** A chimera refers to a hybrid creature composed of the parts of more than one animal. We use the analogy to refer to those read count *observations* for which $(y_{lj} \neq r)$ for all $j = 1, \ldots, S$, or in other words, to those *cell-umi-gene* labels where we observe reads from more than one sample. We can further classify chimeras by the number of collisions. A k-chimera has reads in k sample categories (e.g. $y = (4, 1, 0, 2, 0)$ is a 3-chimera when $S = 5$). In contrast, we refer to an observation with no collisions (i.e. a 1-chimera) as a non-chimera.

If there is no sample index misassignment whatsoever, then we expect that all the M observations to be non-chimeric. When the sample index hopping rate is large, we expect to see a correspondingly large proportion of chimeras, observations where there are collisions of labels across the samples.

Definition 2 (Phantom Molecule)**.** For a given observation with label *cell-umi-gene*, we term molecules observed in samples other than the true source sample as *phantom molecules.* We call them *phantom* since their existence is not real, due only to an artifact brought about by index hopping.

It is important to note that a sample index can hop even when we do not observe a label collision. Thus one or even all reads annotated with a given label may be actually hopping reads originating from the true source sample. In such a case, we are unable to determine the true source sample of these molecules. Nonetheless, we can estimate their expected observed proportions.

Definition 3 (Fugue Observation)**.** A disassociative fugue is a personality disorder characterized by unplanned travel, wandering, and even the establishment of a new identity. Similarly, for a given label, when all the reads hop over from an unobserved sample to establish a new identify in other samples, we term all such observations as *fugues* since we cannot know where they came from since we observe zero reads associated with the true sample of origin.

Definition 4 (Sample Index Hopping Rate)**.** Whereas the term $p_h = \frac{(1-p)}{S-1}$ represents the probability that a sample index hops into a one particular target sample, the complement of p, namely $1 - p$, refers to the quantity of interest, the Sample Index Hopping Rate, which we abbreviate as **SIHR** .

3.2 Estimating SIHR

Given the large number of observations (i.e. number of CUGs M) that are commonly encountered in practice, we proceed to simplify the problem analytically to make computation tractable. In what follows, we reduce the mixture model to a single parameter model for the distribution of non-chimeric observations, we then derive the distribution of the sum of non-chimeric observations and show, after making a third simplifying assumption, that the distribution's mean function corresponds to the solution of a differential equation governing a negative growth process. Then, we show how the index hopping rate can be estimated by formulating the problem as generalized linear regression model for binomial counts with a log link function that corresponds to the solution of the differential equation.

Modeling the Distribution of k-chimeras. The distribution of k-chimeras is essentially the distribution of the total number of non-zero counts of a multinomial random variable. With regards to single cell data, determining whether a sample count is nonzero is equivalent to a deduplication process of obtaining molecule counts from read counts. Statistically, deduplication can be formulated as a thresholded latent variable model where each element of a potentially unobservable random vector $\boldsymbol{y}_l$ is thresholded into an observable Bernoulli random variable z_{ls} with an indicator function $\mathcal{I}$ that detects whether a molecule is observed. That is,

$$\begin{aligned} s_l &\sim \text{Categorical}(\boldsymbol{\pi}_r) \\ \boldsymbol{y}_l | s_l &\sim \text{Multinomial}(r, \boldsymbol{p}_{s_l}) \\ x_{li} &= \mathcal{I}(y_{li} > 0) \; for \; l = 1, \ldots, m_r; i = 1, \ldots, S \end{aligned}$$

where $\boldsymbol{y}_l \in \mathbb{N}_0^S, \; \boldsymbol{p} \in [0,1]^S, \|\boldsymbol{p}\|_1 = 1,$ and $S \in \mathbb{N}$. The marginal distribution of each element of the multinomial observation $\boldsymbol{y}_l$ is binomial.

$$y_{li} | s_l \sim \text{Binomial}(r, p^{[s_l = i]} p_h^{(1-[s_l=i])})$$

where the Iverson bracket notation is used. As we show in Section 3.5, the Bernoulli random variables (i.e. the elements of $\boldsymbol{x}_r$) can be treated as independent (for $r > S$) but not identically distributed, and their sum, which indicates the category of the observation (i.e. $k-$chimera), can be given by the Poisson Binomial distribution.

$$k_r = \mathbf{1}^\intercal \boldsymbol{x}_r \sim PB(\mu_r = \sum_{i=1}^{S} \zeta_i)$$

Modeling the Distribution of Non-chimeras. For the case of $k = 1$, or non-chimeric observations, we can derive a closed form of the distribution by noting that a *non-chimera* is a count observation $\boldsymbol{y}_l$ for which $(y_{li} = r)$ for any $i \in \{1, \ldots, S\}$. We denote the event of observing a *non-chimera* by a Bernoulli random variable $w_l := \mathcal{I}((y_{li} = r))$ with mean parameter given by $p_w = \text{E}(\mathcal{I}((y_{li} = r))$. As a result, the distribution of observing a *non-chimera* is given by

$$w_l | r \sim \text{Bernoulli}\Big(p^r + (S-1) \times \left(\frac{1-p}{S-1}\right)^r\Big)$$

where $p_{f_0}(r) = (S-1) \times \left(\frac{1-p}{S-1}\right)^r$ is the probability of observing a non-chimeric fugue observation with r reads and p^r is the probability of observing a non-chimeric non-fugue observation with r reads.

Modeling the Distribution of Sum of non-Chimeras. Given that p_w is the same for all observations with the same *PCR duplication level*, we can sum all the m_r Bernoulli random variables in *PCR duplication level* r to obtain a Binomial distribution over the number of *non-chimeras* z_r conditional on r. That is, for a given r we have

$$z_r = \sum_{l=1}^{m_r} w_l \sim \text{Binomial}\bigg(m_r,\ p^r + (S-1) \times \left(\frac{1-p}{S-1}\right)^r\bigg)$$

Estimating the Sample Index Hopping Rate. The joint sampling distribution of the non-chimeras at all *PCR duplication level* values, concatenated as a vector $\boldsymbol{z}$, can be decomposed as follows.

$$\text{P}(\boldsymbol{z}|\theta) = \prod_{r=1}^{R} \text{Binomial}\bigg(z_r|m_r,\ p^r + (S-1) \times \left(\frac{1-p}{S-1}\right)^r\bigg) \tag{7}$$

Assumption 3 (The Negligible Contribution of the Probability of Observing non-Chimeric Fugues)**.** Given that the *the sample index hopping rate* we observe in experimental data tends to be very small, $SIHR < 0.05$, the contribution of the second term (i.e. the probability of non-Chimeric fugues) in the parameter of Model 7 is discernible only when $r \leq 2$.

With the aid of Assumption 3, Model 7 can be simplified and the relationship between the number of non-chimeras z_r and the sample index hopping rate $(1-p)$ at a given *PCR duplication level* r and with an m_r number of observations can be formulated as a *generalized linear regression* model with a *log* link function as follows (see Section 3.5 for details).

$$\begin{aligned} z_r &\sim \text{Binomial}\big(z_r|\ N = m_r,\ \mu = e^{\beta r}\big) \\ \textit{where} \quad & z_r, m_r \in \mathbb{N}_+;\ \beta = log(p) \in \mathbb{R}_-;\ r = 2, \ldots R. \end{aligned} \tag{8}$$

An estimate of the sample index hopping rate can be obtained from the regression coefficient.

$$\textbf{SIHR} = 1 - \hat{p} = 1 - exp(\hat{\beta})$$

From which we can obtain the sample barcode index hopping rate.

$$\textbf{SBIHR} = (1 - \hat{p})\Big(\frac{4S-1}{4S-4}\Big)$$

3.3 Inferring Sample of Origin

In Model 6, we denote the true sample of origin of a sample index by the latent variable $\boldsymbol{s_l}$, whose posterior probability distribution can be derived via Bayes theorem. The posterior distribution allows us to quantify our residual uncertainty about the true sample of origin given the observed read count and the set of parameters p and the molecular proportions $\Pi := \{\boldsymbol{\pi}_r\}_{r=1}^{R}$ that govern the mixture model. The posterior distribution of the true sample of origin for each element is given by.

$$\mathbb{P}(\boldsymbol{s_l} = s \mid \boldsymbol{y}_l;\ r, \hat{p}, \hat{\boldsymbol{\pi}}_r) = \frac{\left(\frac{S-1}{\frac{1}{\hat{p}}-1}\right)^{y_{ls}} \hat{\pi}_{rs}}{\sum_{s=1}^{S} \left(\frac{S-1}{\frac{1}{\hat{p}}-1}\right)^{y_{ls}} \hat{\pi}_{rs}}$$

where the plug-in estimates $\hat{\pi}_{rs}$ is the proportion of molecules in sample s at PCR duplication level r as computed by Equation 10 and $\hat{p}$ is the complement of the sample index hopping rate as estimated by Model 8. For derivation, see Section 3.5. We label the sample with the maximum posterior probability as the true sample of origin.

$$s_l^{(t)} = argmax(\mathbb{P}(\boldsymbol{s_l} \mid \boldsymbol{y}_l; r, \hat{p}, \hat{\boldsymbol{\pi}}_r)$$

The corresponding posterior probability of $s_l^{(t)}$ is simply the maximum over the posterior probability vector.

$$q_l|\boldsymbol{y}_l = max(\mathbb{P}(\boldsymbol{s_l} \mid \boldsymbol{y}_l; r, \hat{p}, \hat{\boldsymbol{\pi}}_r)$$

When $\hat{\boldsymbol{\pi}}_r$ is uniform, the maximum $q_l|\boldsymbol{y}_l$ can have duplicated values. However, such an outcome is extremely unlikely given the high variability of the molecular proportions that characterize empirical data and therefore will not be considered.

3.4 Purging Phantoms

For each of the L observations, we label the molecule corresponding to $s_l^{(t)}$ as a real molecule and all the others (with nonzero reads) as phantom molecules. Such a procedure achieves the minimum possible number of false negatives at the expense of false positives. However, it could be well the case that sacrificing a potential real molecule is worth more than retaining a phantom molecule in the dataset. In scientific applications, most often, a weak signal is preferable to an artifactual signal. That said, to achieve the minimum possible number of false positives, it might be the case that the entire data would need to be discarded. An alternative approach attempts to find the optimal trade-off that minimizes both the false positive and false negative rates.

To optimally minimize the false positive rate, we would need to find the optimal cutoff value q^* below which, observations with $s_l^{(t)}$ that we labeled as real molecules are now relabeled as phantom – in other words, we discard them along with previously labeled phantom molecules. In order to determine q^*, we would need to work with the marginal posterior cumulative distribution function of q, which does not have a closed-form expression but which can be expressed as such.

$$\begin{aligned}
\mathbb{P}(q) = P(q|\boldsymbol{y})\mathbb{P}(\boldsymbol{y}) &= \sum_{o \in \wedge^S} \mathbb{P}(q|\boldsymbol{y} = o)\mathbb{P}(\boldsymbol{y} = o) \\
&= \sum_{o \in \mathcal{O}} \sum_{r \in \{1,\ldots,I\}} \mathbb{P}(q|\boldsymbol{y} = o^{(r)})\mathbb{P}(\boldsymbol{y} = o^{(r)}|n = r)\mathbb{P}(n = r) \qquad (9)
\end{aligned}$$

Here, $\mathcal{O}$ consists of all the outcomes $\boldsymbol{y}$ that correspond to the coefficients in r'th component of the Pascal's S-Simplex $\wedge_r^S$. Whereas $\mathbb{P}(\boldsymbol{y} = o^{(r)}|n)$ is given by the multinomial distribution can be computed numerically for the first dozen values of *PCR duplication level* r, the marginal distribution

of r, $\mathbb{P}(n)$, can be approximated by the observed proportion of *PCR duplication levels* which we denoted by $M(.)$ in Model 6. However, since r can have large values in empirical data, we can work with the empirical distribution instead and approximate the marginal distribution of $P(\boldsymbol{y})$ by the observed relative frequency distribution of outcomes (e.g. the proportion of $\boldsymbol{y} = (1, 2, 0, 0)$ observations in the data).

Determining the Optimal False Positive Rate. The approach outlined in the previous section can be thought of as a classification task in which we attempt to predict whether a given molecule is a phantom molecule, which we consequently discard, or a real molecule, in which case we retain. We would like to minimize both the error that a molecule we deem real is in fact a phantom (i.e. false positive) and the error that a molecule we deem phantom is in fact a real molecule (i.e. false negative). In a dataset consisting of L observations and M molecules, the maximum number of real molecules is L. In what follows, it helps to work with proportions relative to the number of observations L. Accordingly, we define $u = \frac{M-L}{L}$ as the *molecule inflation factor*, a lower limit on the fraction of molecules we can predict to be phantom (with respect to L). Although the initial total number of molecules in the entire data before index hopping is L, the upper limit of the number of real molecules we are able to predict as real is slightly less than L due to the existence of fugue observations in the dataset, each one of which would lead to one extra true phantom molecule to be unaccounted for. We term these molecules as *fugue phantoms.* L therefore would equal the sum of real molecules and fugue phantoms. Accordingly, if g is the proportion of fugue observations in the dataset, which we can obtain as shown in Section 3.5, then there would be a total of $(u + g)$ true phantom molecules and a total of $(1 - g)$ true real molecules in the data. For example, for a dataset with $L = 100$ observation, a total number of molecules $M = 150$, and an estimated proportion of fugues $g = 0.05$, the *molecule inflation factor* u would equal 0.5 so that the total number of true phantom molecules is given by $(0.5 + 0.05) \times L = 55$ and the total number of real molecules is given by $(1 - 0.05) \times L = 95$.

Given that the number of phantom molecules in a given dataset is given by $L \times (u + g)$, there are three courses of action we can choose to take.

1. *No Purging*: We keep the data as it is. By doing so, we basically label all the phantom molecules as real, which would correspond to $L \times (u + g)$ false positives (i.e. FPR=1). That is, we are in effect incorrectly classifying all true phantom molecules as real.

2. *No Discarding*: We purge the phantom molecules by reassigning the reads to the sample with largest posterior probability. By doing so, we can drastically decrease the number of false positives while incurring relatively small number of false negatives.

3. *Discarding Below Cutoff*: We can decrease the false positives further at the cost of a slight marginal increase in false negatives by choosing a cutoff $q*$ below which predicted real molecules are classified as phantom instead. That is, in our classification task, we label the molecule with the maximum posterior probability (q) above or equal to a given threshold $q*$ as a real molecule while the remaining molecules are all labelled as phantoms. Furthermore, all the molecules in observations whose corresponding q falls below the cutoff are also labeled as predicted phantoms even though a proportion of them are real molecules which we cannot confidently classify (i.e. false negatives). Effectively, all molecules we label as phantom get eliminated from the dataset.

In what follows, it would be easier to work with the complement of q, which we denote $qr = 1 - q$, which is the probability of a molecule originating from a sample other than the one with maximum

posterior probability. For a given selected threshold value qr^*, we let $o^* = F_{qr}(qr^*) := \Pr(qr \leq qr^*)$ denote the proportion of observations whose corresponding predicted real molecules we retain. Here, F_{qr} is the empirical CDF of qr. Consequently, the fraction of predicted real molecules is o^* and the fraction of predicted phantom molecules is $1 + u - o^*$, since the total fraction of molecules must sum up to $u + 1$ (see **Supplementary Table 2**). Out of the (o^*) predicted real molecules, the proportion of false positives is given by the expectation of the probability of false assignment over the subset of the data o^*.

$$FP(qr^*) = \int_0^{qr^*} 1 - F_{qr}(qr) \ \mathrm{d}(qr) = \int_0^{qr^*} qr \ f_{qr}(qr) \ \mathrm{d}(qr).$$

Note that the empirical cumulative distribution function of qr is discrete (i.e. its ECDF increases by jump discontinuities only) even though it takes real values in $\mathbb{R}^{[0,1]}$. Accordingly, the threshold qr^* should be set such that very few potential real molecules are discarded for any marginal decrease in false positives.

The TOR approach. The optimal value for the threshold qr^* is the one that simultaneously minimizes both the number of FP counts and FN counts, but note that discarding lower quality data would lead to a small FP but also a large FN. Although we can use *Youden's* **J** statistic = 1-(FPR +FNR) to find the optimal cut-off value $qr*$ that maximizes **J**, where (FPR) := $\frac{FP}{(u+g)}$ and FNR := $\frac{FN}{(1-g)} = \frac{o^*+FP-g}{(1-g)}$, the measure is not appropriate for our application (see Section 3.5). An alternative approach for minimizing both the number of FP counts and FN counts considers the ratio of marginal increase in FNs over the marginal decrease in FNs as a trade-off to be specified by the researcher, the Trade-Off Ratio cutoff *TOR* parameter, which represents the maximum number of real molecules one is willing to incorrectly discard in order to correctly purge one phantom molecule. That is, we make our reference coordinates (the point of origin) to be the number of false positive and false negative molecules we obtain if we reassign reads without purging any predicted real molecules. As we discard an increasing number of predicted real molecules, the marginal false positive (i.e. predicted real molecules that are actually phantom) decreases while the marginal false negative (i.e. real molecules that we effectively predict as phantom by discarding) increases. For a given dataset, the cutoff *TOR** that gets effectively chosen would correspond to the largest observed *TOR* value not exceeding the preset *TOR* cutoff value. All molecules with corresponding *TOR* values strictly less than *TOR* cutoff* - not *TOR cutoff*- are discarded. For example, if we have $tor = (0.1, 0.5, 2.9, 4.1, \ldots)$ and *TOR cutoff*=3, then *TOR* cutoff* =2.9 and predicted real molecules corresponding to $tor = 0.1$ and $tor = 0.5$ are discarded. **Supplementary Figure 3** left shows the trade-off between false negatives and false positives for all four datasets. As can be seen, the optimal cutoff occurs at the point that is closer to the origin. Note that a small marginal decrease in FPs can be offset only by a large increase in FNs. **Supplementary Figure 4** right shows the marginal increase in false negatives against the marginal decrease in false positives with the default *TOR* value in color.

Example: Consider the table below. This table shows the *TOR* cutoff values, corresponding outcome, inferred sample of origin s, posterior probability qr, false positive (FP), and false negative (FN) counts. Values corresponding to no purging, no discarding, and to the next more conservative *TOR* cutoffs are listed for the mouse epithelial non-multiplexed HiSeq 2500 and multiplexed HiSeq 4000 datasets and the two lanes of the Tabula Muris NovaSeq 6000 datasets.

dataset	approach	outcome	s	qr	TOR	FP	FN
HiSeq2500	above	0,0,1,0,1,0,0,0	5	0.00630	49.64	984	23,384
HiSeq2500	TOR	0,0,1,0,0,0,0,0	3	0.00731	2.97	1147	1201
HiSeq2500	nodiscarding	1,0,0,0,0,0,1,0	7	0.49826	0.00	1,449	303
HiSeq2500	nopurging					27,393	0
HiSeq4000	above	0,1,2,1,1,0,0,0	3	0.10741	6.24	69,241	114,007
HiSeq4000	TOR	0,0,1,0,0,0,0,0	3	0.11089	2.96	80,320	25,186
HiSeq4000	nodiscarding	0,1,0,0,1,0,1,0	5	0.56309	0.00	86,289	7,546
HiSeq4000	nopurging					4,460,074	0
NovaSeqL1	above	0,0,2,0,0,0,0,0,0,0,2,0,0,0,0,0	3	0.07375	3.01	558,115	623,953
NovaSeqL1	TOR	0,0,0,0,0,0,0,0,0,0,1,1,0,0,0,0,0	10	0.07399	2.99	558,550	618,509
NovaSeqL1	nodiscarding	0,0,0,0,1,0,1,0,1,1,0,0,1,0,0,0	10	0.72582	0.00	712,958	157,145
NovaSeqL1	nopurging					11,711,946	0
NovaSeqL2	above	0,0,1,0,0,0,0,0,0,0,1,0,0,0,1,0	3	0.07390	3.01	568,394	631,097
NovaSeqL2	TOR	0,0,0,0,0,0,0,0,0,0,1,1,0,0,0,0,0	10	0.07393	2.98	568,846	625,445
NovaSeqL2	nodiscarding	0,0,0,1,0,0,0,0,1,1,0,1,0,1,0,1	10	0.76330	0.00	725,117	159,048
NovaSeqL2	nopurging					11,902,481	0

As evident from this table, leaving the data as it is (i.e. *nopurging*) comes at cost in the form of a large number of false positives (i.e. true phantom molecules that we incorrectly label as real molecules). In contrast, by reassigning reads to the sample with the maximum posterior probability and retaining only those associated molecules (i.e. *nodiscarding*), we are able to substantially reduce the number of false positives at a very little cost of removing true real molecules. We can further minimize the number of false positives by discarding predicted real molecules that have low posterior probability. By using the default cutoff $TOR = 3$ value, we retain molecules with TOR values that are equal or greater than the maximum TOR value not exceeding the cutoff $TOR = 3$. In the table, the rows corresponding to the *above* approach shows the corresponding outcome, inferred sample of origin s, posterior probability qr, false positive (FP) and false negative (FN) counts, and the TOR value for the observed outcome with the next higher TOR value, the one with a lower false positive count.

3.5 Mathematical Derivations

The Molecular Proportions Complexity Profile. Let $\boldsymbol{v}_r = \sum_l^{m_r} \boldsymbol{s}_l$ denote the molecule count across the S samples at level r and $\boldsymbol{v} = \sum_r^R \sum_l^{m_r} \boldsymbol{v}_r = \sum_r^R \sum_l^{m_r} \boldsymbol{s}_l$ the molecule count across the S samples for the entire data. Since $\mathrm{E}(\boldsymbol{v}_r) = m_r \times \boldsymbol{\pi}_r$, the expected total molecule count is therefore $\mathrm{E}(\boldsymbol{v}) = \sum_r^R m_r \times \boldsymbol{\pi}_r$. If $\boldsymbol{\pi}_r = \boldsymbol{\pi}$ for all $r = 1, \ldots, R$, then $\mathrm{E}(\boldsymbol{v}) = L \times \boldsymbol{\pi}$ would correspond to the *library complexities* of the S samples.

Let $\boldsymbol{u}_r = (u_{r1}, \ldots u_{rS})(= \sum_l^{m_r} \boldsymbol{s}_l)$ represent the unobserved molecule (i.e. UMIs) counts at *PCR duplication level* r across S samples. The distribution of $\boldsymbol{u}_r$ can be obtained by noting that sum of m_r identically distributed categorical random variables with parameter vector $\boldsymbol{\pi}_r$ is a multinomial random variable with the same parameter vector. That is,

$$\boldsymbol{u}_r = \sum_l^{m_r} \boldsymbol{s}_l \sim \text{Multinomial}(m_r, \boldsymbol{\pi}_r)$$

Now, the expected molecule count across samples in the entire data is given as such.

$$\mathrm{E} \sum_{r \in \mathcal{R}} \boldsymbol{u}_r = \sum_{r \in \mathcal{R}} (m_r \times \boldsymbol{\pi}_r)$$

However, we do not observe $\boldsymbol{s}_l$, and the expected total number of observed molecules in the data can be much greater due to the presence of phantom molecules brought about by sample index hopping.

Let $\boldsymbol{v}_r = \sum_l^{m_r} \boldsymbol{y}_l$ denote the total read count at *PCR duplication level* r. In what follows, it helps to think of the m_r observations as members of S partitions, where the size of partition s is given by π_{rs}, the proportion of molecules in Sample s at *PCR duplication level* r. Now since

$$\begin{aligned}
\mathrm{E}(\boldsymbol{v}_r) &= \mathrm{E}(\sum_l^{m_r} \boldsymbol{y}_l) = \sum_l^{m_r} \mathrm{E}_s(\mathrm{E}_{y|s}(\boldsymbol{y}_l)) \\
&= (m_r \times r) \sum_{s=1}^{S} \boldsymbol{p}_s \pi_{rs}
\end{aligned}$$

therefore

$$\mathrm{E}(v_{rs}) = (m_r \times r)\Big(\frac{\pi_{rs}(S \times p - 1) + (1 - p)}{S - 1}\Big)$$

and so we can obtain an estimate for π_{rs} from the observed proportion of read counts $\bar{v}_{rs}$ for sample s observations at *PCR duplication level* r. That is,

$$\hat{\pi}_{rs} = \frac{\bar{v}_{rs}(S - 1) + (p - 1)}{(S \times p - 1)} \tag{10}$$

Notice that when there is no sample index hopping (i.e. $p = 1$), the proportion of molecules equals the proportion of reads, that is $\hat{\pi}_{rs} = \bar{v}_{rs}$. Also, note that for the denominator to be positive, the proportion of reads $\bar{v}_{rs} > \frac{1-p}{S-1}$ for all $s = 1, \ldots, S$. In empirical data, when this relationship is violated, we can set $\hat{\pi}_{rs} = 10^{-6}$ and renormalize $\hat{\boldsymbol{\pi}}_r$ accordingly.

The Distribution of k-chimeras. The sampling distribution of observing a molecule in sample i is

$$\begin{aligned}
s_l &\sim \text{Categorical}(\boldsymbol{\pi}_r) \\
\boldsymbol{y}_l | s_l &\sim \text{Multinomial}(r, \boldsymbol{p}_{s_l}) \\
x_{li} &= \mathcal{I}(y_{li} > 0) \ \textit{for}\ l = 1, \ldots, m_r; i = 1, \ldots, S
\end{aligned}$$

where $\boldsymbol{y}_l \in \mathbb{N}_0^S,\ \boldsymbol{p} \in [0,1]^S, \|\boldsymbol{p}\|_1 = 1,$ and $S \in \mathbb{N}$. Now, the marginal distribution of each element of the multinomial observation $\boldsymbol{y}_l$ is binomial. That is,

$$y_{li}|s_l \sim \text{Binomial}(r, p^{[s_l=i]} p_h^{(1-[s_l=i])})$$

where the Iverson bracket notation is used. It follows that the distribution of x_{li} is a Bernoulli with a probability parameter that can be obtained by observing that the expectation of an event defined by an indicator function is equal to the probability of that event. That is,

$$x_{li} \sim \text{Bernoulli}(\zeta_i)$$

where

$$
\begin{aligned}
\zeta_i &= \mathrm{E}(x_{li}) = \mathrm{E}_s(\mathrm{E}_{x|s}(x_{li})) \\
&= \mathrm{E}_s(\mathrm{E}_{x|s}(\mathcal{I}(y_{li} > 0))) \\
&= \sum_{j=1}^{S} \mathbb{P}(y_{li} > 0 | s_l = j) \, \mathrm{P}(s_l = j) \\
&= 1 - \sum_{j=1}^{S} \mathbb{P}(y_{li} = 0 | s_l = j) \, \mathrm{P}(s_l = j) \\
&= 1 - \sum_{j=1}^{S} \left(1 - p^{[j=i]} p_h^{(1-[j=i])}\right)^r \pi_j^{(r)} \\
&= 1 - \left(\pi_i^{(r)}(1-p)^r + (1-\pi_i^{(r)})((1-p_h)^r)\right) \\
&= 1 - \pi_i^{(r)} - \left(\left(\frac{1}{1-p} - \frac{1}{S-1}\right)^r \left((1-p)^r - \pi_i^{(r)}\right)\right)
\end{aligned}
$$

Now given that the Bernoulli random variables (i.e. the elements of $\boldsymbol{x}_r$) can be treated as independent (for $r > S$) but not identically distributed, their sum, which indicates the category of the observation (i.e. $k-$chimera), can be given by the Poisson Binomial distribution. That is,

$$k_r = \mathbf{1}^\intercal \boldsymbol{x}_r \sim PB(\mu_r = \sum_{i=1}^{S} \zeta_i)$$

and a PMF given by the following recursive formula $\boldsymbol{\zeta}$

$$
\Pr(K = k) = \begin{cases}
\prod_{i=1}^{S} (1 - \zeta_i) & k = 0 \\
\frac{1}{k} \sum_{i=1}^{k} (-1)^{i-1} \Pr(K = k - i) T(i) & k > 0 \\
where \;\; T(i) = \sum_{j=1}^{S} \left(\frac{\zeta_j}{1-\zeta_j}\right)^i
\end{cases}
$$

Note the independence assumption does not hold when $r < S$ since $\Pr(K = 0)$ is not zero even though it should be given that the r reads must be belong to at least one sample. Also note that the mean, μ_r, of the Poisson Binomial distribution is equal to the sum of the probabilities. In this case, the $\pi_i^{(r)}$'s do not affect μ_r since they cancel out. That said, they do affect the variance $\sigma_l^2 = \sum_{i=1}^{n} (1 - \zeta_i)\zeta_i$ where it is maximized when the ζ_i's, and accordingly the $\pi_i^{(r)}$'s, are equal. In this case k_l can be reduced to the sum of dependent Bernoulli variables, which for $r > S$ can be approximated as the sum of independent and identically distributed Bernoulli variables. That is,

$$k_r \sim \text{Binomial}\left(S, 1 - \frac{1}{S}\left((1-p)^r + (S-1)^{1-r}(S + p - 2)^r\right)\right)$$

The distribution of phantoms can be obtained from k_r by noting that for non-fugue observations, the number of phantoms equals $k_r - 1$. The expected fraction of phantoms at *PCR duplication level* r is therefore approximately as such.

$$\mathrm{E}(f_l) = S - 1 - (1-p)^r - (S-1)^{1-r}(S+p-2)^r \tag{11}$$

The figure below plots the expected fraction of phantoms as a function of the *PCR duplication level* r for a range of values of p and number of samples S:

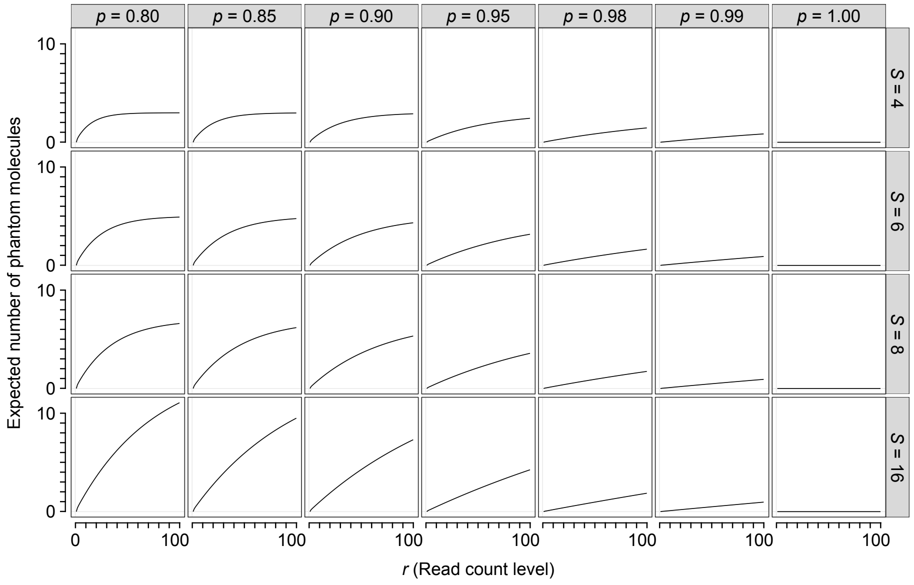

Distribution of non-chimeras

For the case of $k = 1$, or non-chimeric observations, we can derive a closed form of the distribution by noting that a *non-chimera* is a count observation $\boldsymbol{y}_l$ for which $(y_{li} = r)$ for any $i \in \{1, \ldots, S\}$. We denote the event of observing a *non-chimera* by a Bernoulli random variable $w_l := \mathcal{I}((y_{li} = r))$ with parameter p_w given by

$$\begin{aligned}
p_w &= \mathrm{E}(\mathcal{I}((y_{li} = r)) = \mathrm{P}(y_{lji} = r) \\
&= \sum_{j=1}^{S}\sum_{i=1}^{S} \mathrm{P}(y_{li} = r|(s_l = j))\,\mathrm{P}(s_l = j) \\
&= \sum_{j=1}^{S}\sum_{i=1}^{S} \left(p^{[j=i]} p_h^{(1-[j=i])}\right)^r \pi_j^{(r)} \\
&= \sum_{j=1}^{S} [p^r + (S-1) \times \left(\frac{1-p}{S-1}\right)^r] \pi_j^{(r)} \\
&= p^r + (S-1) \times \left(\frac{1-p}{S-1}\right)^r
\end{aligned}$$

As a result, the distribution of observing a *non-chimera* is given by

$$w_l|r \sim \text{Bernoulli}\Big(p^r + (S-1) \times \left(\frac{1-p}{S-1}\right)^r\Big)$$

where $p_{f_0}(r) = (S-1) \times \left(\frac{1-p}{S-1}\right)^r$ is the probability of observing a non-chimeric fugue observation with r reads and p^r is the probability of observing a non-chimeric non-fugue observation with r reads.

Estimation of the Sample Index Hopping Rate

$$\begin{aligned} \mathrm{P}(\boldsymbol{z}|\theta) &= \prod_{r=1}^{R} \mathrm{P}(z_r|m_r, \mu(p,r)) \\ &= \prod_{r=1}^{R} \text{Binomial}\big(z_r|m_r,\ p^r\big) \end{aligned}$$

Note that the mean function $\mu(r) = p^r$ can be expressed as such.

$$\mu(r) = p^r = e^{r\ log(p)} = e^{\beta r}$$

which corresponds to the integral curve solution of the differential equation

$$\frac{d\mu(r)}{d\ r} = \beta\mu(r)$$

This is a negative growth curve since $\beta = log(p) \in (-\infty, 0)$. We can estimate β by formulating the problem as a generalized linear regression for binomial counts with a systematic component $\eta = \beta r$ and a log link function $g(\mu) = log(\mu)$ such that.

$$\mu = g^{-1}(\eta) = e^{\eta}$$

Note that the standard link function for binomial counts is the *logit* $g(\mu) = log(\frac{\mu}{1-\mu})$. However, it corresponds to the solution of another differential equation, namely.

$$\frac{d\mu(r)}{dr} = \beta\ \mu(r)\big(1-\mu(r)\big)$$

Although the log link function is not symmetric, η is defined only on the negative real line. Furthermore, the *log* link renders the parameters interpretable since β is basically just the log of p the parameter of interest.

The relationship between the number of non-chimeras z_r and the sample index hopping rate $(1-p)$ at a given *PCR duplication level* r and with an m_r number of observations can be formulated as a *generalized linear regression* model with a *log* link function as follows.

$$\begin{aligned} z_r &\sim \text{Binomial}\big(z_r|\ N = m_r,\ \mu = e^{\beta r}\big) \qquad (12) \\ \textit{where} \quad & z_r, m_r \in \mathbb{N}_+;\ \beta = log(p) \in \mathbb{R}_-;\ r = 2, \ldots R. \end{aligned}$$

An estimate of the sample index hopping rate can be obtained from the regression coefficient as such.

$$\begin{aligned}\textbf{SIHR} &= 1 - \hat{p} \\ &= 1 - exp(\hat{\beta})\end{aligned}$$

We can use the estimate of the sample index hopping rate to obtain the expected number of hopped reads at each *PCR duplication level*, namely ($m_r \times r \times \textbf{SIHR}$). We can also plug it into Equation 11 to compute the expected Fraction of Phantom Molecules (**FPM**) for the entire dataset. That is.

$$\begin{aligned}\textbf{FPM} &= \text{E}_m(\text{E}(f_r)) \\ &= \frac{\sum_{r=1}^{R} m_r \times \text{E}(f_r)}{\sum_{r=1}^{R} m_r} \\ &= \frac{\sum_{r=1}^{R} m_r \times \Big(S - 1 + (1-\hat{p})^r + (S-1)^{r-1}(S + \hat{p} - 2)^r\Big)}{\sum_{r=1}^{R} m_r} \\ &= \boldsymbol{m}_p^{\intercal}\, \boldsymbol{f}\end{aligned}$$

Here $\boldsymbol{m}_p = \frac{\boldsymbol{m}}{\|\boldsymbol{m}\|_1}$ where $\boldsymbol{m} = (m_1, \ldots, m_R)$ and $\boldsymbol{f} = (\text{E}(f_1), \ldots, \text{E}(f_R))$.

We would like to note that we can do away with Assumption 3 and estimate p using a likelihood optimization procedure. However, the optimization algorithm might not converge or it can get stuck at a local optimum. Furthermore, compared to the GLM approach, it is not straightforward to obtain standard errors or any measure of uncertainty by optimizing the likelihood. We provide code in the paper's GitHub repository showing the likelihood optimization approach and its overall similar results to the GLM approach.

Estimating the Sample Barcode Index Hopping Rate

A sample in a 10X Genomics experiment is actually labelled by, not one, but four different barcodes. By considering only the sample labels instead of the sample barcode, the statistical approach outlined in this paper models sample index hopping across samples rather than across individual barcodes. Nonetheless, we can still estimate the sample barcode index hopping rate by noting that when we treat all reads of a given sample as having the same sample index, we are effectively collapsing a $4 \times S$-dimensional multinomial vector of barcode sample index hopping probabilities $\boldsymbol{p}_b$ into an S-dimensional vector of sample index hopping probabilities $\boldsymbol{p}$. That is, since the read counts are modeled with a multinomial, we can fuse the four barcode count events within a sample by summing their read counts and corresponding probabilities. Accordingly, the relationship between the two parameters of interest p_b and p can be expressed as such.

$$p = p_b + \frac{3(1-p_b)}{4S-1} = p_b\Big(1 - \frac{3}{4S-1}\Big) + \frac{3}{4S-1}$$

where p_b is probability that a given sample barcode index does not hop. Accordingly, we can obtain an estimate of the sample barcode index hopping rate by expressing p_b in terms of p and S.

$$\mathbf{SBIHR} = 1 - p_b = (1-p)\Big(\frac{4S-1}{4S-4}\Big)$$

Notice that the multiplicative factor is at its maximum (i.e. 1.75) when $S = 2$. As S increases however, it approaches 1 and the two terms become approximately equal.

The Posterior Distribution of the True Sample of Origin

The posterior distribution can be obtained as follows.

$$\begin{aligned}
\mathbb{P}(\boldsymbol{s_l} \mid \boldsymbol{y}_l;\ r, \hat{p}, \hat{\boldsymbol{\pi}}_r) &= \frac{\mathbb{P}(\boldsymbol{y}_l \mid \boldsymbol{s_l};\ r, \hat{p})\mathbb{P}(\boldsymbol{s_l}; \hat{\boldsymbol{\pi}}_r)}{\mathbb{P}(\boldsymbol{y}_l)} \\
&= \frac{\text{Multinomial}(\boldsymbol{y}_l \mid \boldsymbol{s_l}; r, \hat{\boldsymbol{p}}_{s_l})\ \text{Categorical}(\boldsymbol{s_l}; \hat{\boldsymbol{\pi}}_r)}{\sum_{s=1}^{S} \text{Multinomial}(\boldsymbol{y}_l \mid \boldsymbol{s_l}; r, \hat{\boldsymbol{p}}_{s_l})\ \text{Categorical}(\boldsymbol{s_l}; \hat{\boldsymbol{\pi}}_r)} \\
&= \frac{\left(\hat{\boldsymbol{p}}_{s_l}^{\boldsymbol{y}_l}\right)\left(\hat{\boldsymbol{\pi}}_r^{\boldsymbol{s}_l}\right)}{\sum_{s=1}^{S}\left(\hat{\boldsymbol{p}}_{s_l}^{\boldsymbol{y}_l}\right)\left(\hat{\boldsymbol{\pi}}_r^{\boldsymbol{s}_l}\right)}
\end{aligned}$$

which for each element, simplifies to

$$\mathbb{P}(\boldsymbol{s_l} = s \mid \boldsymbol{y}_l;\ r, \hat{p}, \hat{\boldsymbol{\pi}}_r) = \frac{\left(\frac{S-1}{\frac{1}{\hat{p}}-1}\right)^{y_{ls}} \hat{\pi}_{rs}}{\sum_{s=1}^{S}\left(\frac{S-1}{\frac{1}{\hat{p}}-1}\right)^{y_{ls}} \hat{\pi}_{rs}}$$

Youden's J statistic.

The cut-off given by *Youden's* **J** is optimal in the sense that it minimizes the probability of random guessing when we give equal weight to FPR and FNR, or equivalently to sensitivity and specificity [11]. Nonetheless, optimizing **J** might not be the most appropriate choice for the problem at hand since the number of phantom molecules is an order of magnitude smaller than the number of real molecules. That is, FPR and FNR are proportions of unbalanced classes, whose denominators depend on u, which is a function of the hopping rate, and g, which not only depends on the hopping rate, but whose estimate can be biased.

Computing the Proportion of Fugue Observations (g)

To obtain the proportion of all fugues $\mathbf{g}$, we need to take into account the proportion of chimeric fugues $\mathbf{g_c}$ as well the proportion of non-chimeric fugues, which we denote by $\mathbf{g_0}$. We can compute $\mathbf{g_0}$ by multiplying the observed relative frequency proportion vector of the read count levels (i.e. $\overline{\boldsymbol{m}}$) by the vector of non-chimeric fugue probabilities, whose elements are given by $p_{f_0}(r)$.

$$\begin{aligned}
\mathbf{g_0} = \mathrm{E}_r\left(p_{f_0}(r)\right) &= \sum_{r=1}^{R} \overline{m}_r \times (p_{f_0}(r)) \\
&= \sum_{r=1}^{R} \overline{m}_r \times (S-1) \times \left(\frac{1-p}{S-1}\right)^r \\
&= \overline{\boldsymbol{m}}^\intercal\ \boldsymbol{p_{f_0}} \qquad (13)
\end{aligned}$$

The probability of observing chimeric fugues can be obtained as such.

$$p_{f_c}(r) = \sum_{\boldsymbol{y}\in\mathcal{T}_r} \binom{r}{\boldsymbol{y}} \times \left(\frac{1-p}{S-1}\right)^r \tag{14}$$

where the set $\mathcal{T}_r$ consists of outcomes corresponding to events where all the r reads hopped to two or more target samples: the outcomes (0, 2, 1) and (0, 1, 2) in **Supplementary Figure 8**. For $r = 1$, the set $\mathcal{T}_r$ is empty; for $r = 2$, there are $\binom{S-1}{r} = \frac{(S-1)(S-2)}{2}$ such outcomes. For $r > 3$, it becomes increasing hard to enumerate and compute the probabilities for all the possible outcomes, especially when S is large as well. However, the value of $\left(\frac{1-p}{S-1}\right)^r$ becomes negligibly small for $r > 3$ given that p tends to be very close to 1. Furthermore, empirical data is characterized by a highly skewed distribution of *PCR duplication levels* such that the proportion of observations in the first few r values makes up a substantial fraction of all observations. Therefore even if $p_{f_c}(r)$ was not negligible for $r > 3$, it would ultimately be weighted down by the respective $\overline{m}_r$. Under this assumption, the proportion of fugue observations can therefore be accurately approximated as such.

$$\begin{aligned}
\mathbf{g} &= \mathbf{g_0} + \mathbf{g_c} \\
&= \sum_{r=1}^{R} \overline{m}_r \times \big(p_{f_0}(r) + p_{f_c}(r)\big) \\
&\approx \overline{m}_1 (S-1)\big(\frac{1-p}{S-1}\big) \\
&\quad + \ \overline{m}_2\Big((S-1)\big(\frac{1-p}{S-1}\big)^2 + 2\frac{(S-1)(S-2)}{2}\big(\frac{1-p}{S-1}\big)^2\Big) \\
&\quad + \cdots \\
&= \overline{m}_1(1-p) + \overline{m}_2(1-p)^2 + \overline{m}_3(1-p)^3 + \cdots
\end{aligned} \tag{15}$$

For example when $p = .99$, $\overline{m}_1 = 0.20$, $\overline{m}_2 = 0.15$, and $\overline{m}_3 = 0.1$ $\mathbf{g} = 0.0020151$. Or in other words, only 0.2 % of observations are fugues. If all the data is restricted to have *PCR duplication level* $r = 1$ (i.e. $\overline{m}_1 = 1$), then $\mathbf{g} = (1-p)$ would be the maximum possible proportion of fugue observations.

Supplementary Discussion

4.1 Limitations of the MRF Approach

The computational strategy proposed in the paper [5] is the only attempt we are aware of that is aimed at estimating the rate and mitigating the effects of index hopping in droplet-based scRNA-seq data. Nonetheless, the strategy the authors propose, does not in fact estimate the index hopping rate of individual reads, but rather computes a proxy measure, *the swapped fraction*, which they define as the fraction of molecules (i.e. those with an identical UMI, cell barcode, gene label) that are observed in more than one sample [5]. As we show in this paper, the probability at which an individual sequencing read swaps the sample index is not the same as the probability that one out of all the PCR-duplicated reads from the same molecule swaps the sample index. As we show, even when the sample index hopping probability at the level of individual read is very low, the fraction of *phantom molecules*, molecules that we observe to have swapped sample indices, can vary greatly, even as far as comprising the vast majority of molecules in a given sample. As for correcting for the effects of index hopping, the proposed molecular exclusion heuristic the authors propose suffers from high false negative rates, resulting in unnecessarily discarding molecules that otherwise should have been retained. In particular, by assigning the true sample of origin as the sample with the largest read fraction while discarding all molecules that have a read fraction below a given threshold (default being ≤ 0.8), the approach effectively discards information that is implicit in the absolute read count distribution and more importantly the extremely variable distribution of molecular proportions (i.e. *library complexity*) across samples and *PCR duplication levels*. For example, even if an observation such as $\boldsymbol{y} = (1, 4, 0, 0)$ has the highest read fraction at $s = 2$, it would still be an unlikely event if for instance we have 70 times more molecules in Sample 1 than in Sample 2, that is, $\pi_5 = (0.7, 0.01, 0.19, 0.1)$.

Contrasts with Proposed Approach. The probabilistic model we propose in this paper aims at capturing the distributional patterns of sequencing read counts in order to both estimate the fundamental quantity of interest *the sample barcode index hopping rate* at the level of individual reads and quantify its manifestation as a data contamination artifact as measured by *the fraction of phantom molecules*. As we show (see Equation 11), the fraction of phantom molecules is determined not only by the sample index hopping rate, but also by the number of multiplexed samples, as well as by their library size, coverage, and molecular proportions complexity profiles (i.e. the distribution of molecular proportions conditional on the *PCR duplication levels*). As such, this complex relationship, which we have attempted to capture in our proposed model, can potentially provide an explanation for the large variance in the index hopping rate estimates (0.2% - 8%) that have been reported in the literature [5,6,8,9]. Furthermore, we use the probabilistic modeling approach to discard molecules not based on their observed read fraction, but rather on a corresponding posterior probability that is a function of not only the distribution of the read counts, but also the model's estimated sample index hopping rate, the library sizes and the expression profiles of the multiplexed samples, as the following paragraph illustrates.

Illustrative Examples from Empirical Data Consider the following scenarios encountered in the data. For example, in the HiSeq 4000 dataset, q (the maximum posterior probability of the true sample of origin) is higher for outcome $y^{(opt)} = (0, 0, 0, 1, 1, 0, 0, 1)$ than it is for $y^{(below)} = (0, 1, 2, 0, 0, 0, 1, 0)$ even though in the latter, the inferred true sample of origin $s = 3$ (Sample B1) has two reads whereas the three molecules in the former has one read each, including the inferred true sample of origin $s = 5$. This might seem counter-intuitive at first sight, but once we

consider the proportion of molecules, it becomes apparent why it makes sense to retain the molecule corresponding to $s = 5$ but discard the one corresponding to $s = 3$. For this observation, we have $\hat{\pi}_r = (0.075, 0.136, 0.004, 0.005, 0.169, 0.517, 0.082, 0.011)$ for $r = 3$ for $r = 4$. Given that the lowest proportions are for $s = 3, 4, 8$ in that order, and highest for $s = 5$, it does make sense indeed to expect that the two reads $y^{(opt)}$ originated from $s = 5$. In contrast, for $y^{(below)}$, it is not really clear that the hopped reads originated from $s = 3$ given the sample has the lowest proportion of molecules. For the HiSeq 2500 data, we have $\hat{\pi}_r = (0.096, 0.173, 0.001, 0.001, 0.216, 0.409, 0.093, 0.011)$ for $r = 3$, which explains why we end up discarding the molecule $s = 8$ in $y^{(below)}$ but not in $y^{(opt)}$ since there are half the number of molecules in $s = 5$ than in $s = 6$, making it more likely that the read indeed hopped from $s = 8$. We have a similar situation for the NovaSeq 6000 datasets where the sample with the lowest proportion of molecules (i.e. 0.005) corresponds to $s = 15$ (i.e. Sample $P7\_8$), making it more likely that the two hopped reads in $y^{(opt)}$ of L1, the one hopped read in in $y^{(below)}$ of L1, and the one hopped read in $y^{(opt)}$ of L2 actually originated from $s = 9$. This also makes $y^{(below)}$ of L2 more likely to be a fugue non-chimeric observation (see definitions, Methods Section), such that the one read in $s = 15$ would in reality have had hopped from a sample with a larger number of molecules.

4.2 Limitations of our model-based approach

Departures from model's assumptions. As **Supplementary Figure 2** shows, the observed chimeric proportions for the experimental datasets show a downward deviation from the GLM predicted mean trend at high *PCR duplication levels*, especially for those top 1 percent of observations that are characterized by noisiness and sparsity. In fact, the downward trend tends to occur right after the cumulative proportion of molecules in the dataset begins to plateau. The downward trend we observe in the proportion of chimeras seems to indicate a slight decrease in hopping rate that kicks in at high PCR duplication levels. This deviation could potentially be explained by sample dropout at high r values. That is, when we no longer observe molecules for one or more samples at high PCR duplication levels. When this is the case the multinomial model we specified can no longer assume that we have S samples since sample indices cannot hop to nonexistent sample molecules. Accordingly, we expect the curve to differ when we have S samples compared to when we have fewer. Furthermore, at very high r values, the observed trend also becomes noisy and fluctuating due to the high proportion of missingness. That is, when $r > 25$, where the number of potential outcomes are in the millions, there would be a finite number of observations at a given r, and not enough to cover all possible outcomes. When this happens, the variability of the mean estimate would increase as the number of observations at a given r decreases. As for the validation dataset, we notice two trends that seem to slightly depart from the model's assumption. First, there is a minor difference between the two samples' proportion of hopped reads, $SIHR_1 = 0.00346$ vs $SIHR_2 = 0.00302$ (the subscript denotes the source sample). Second, the ground truth estimates for both samples start out at higher proportion values but stabilize starting at $r = 10$ (see the reproducible R markdown notebook *validation_hiseq4000_2.nb.html* for details). These trends could be sub-sampling artifacts of the filtering procedure step in which we only retained CUGs that are observed in both the multiplexed and non-multiplexed samples (each of which has twice the number of reads). Alternatively, these trends could be persistent across experiments in which case the model we have proposed here captures rather well, but not perfectly, the underlying mechanism of sample index hopping. A model that is governed by several sample-specific hopping rates that are also dependent on the number of duplicated reads would provide better accuracy but at the cost of intractable computational and mathematical complexity. Even then, the improved accuracy in estimating the hopping rate would not affect the purging procedure greatly given that the molecular complexity profiles plays an important role in

assigning reads to their sample of origin.

The non-negligible probability of a *cell-umi-gene* label collision across samples when the number of samples is large. As the number of multiplexed samples increases, Corollary 1 would no longer hold since the probability of observing, in more than one sample, a given *cell-umi-gene* label combination becomes non-negligible. That said, in single cell experiments, multiplexing more than 16 samples on a single lane is not commonly done given the smaller library size and lower genomic coverage that follows as a result. Furthermore, the adoption of a longer UMI index in the latest 10X Genomics Single Cell 3' v3 assay would further reduce the probability of potential collisions, thus rendering this concern less of a problem.

The sensitivity of the FPR-minimizing cutoff on the ECDF of q. As we have already mentioned, the marginal distribution of q given by Equation 9 has no closed-form nor is it feasible to compute the exact probabilities for all its possible outcomes even when r is not large. As r increases, we tend to observe in the data an increasingly fewer proportion of all possible outcomes and subsequently we would expect the resulting ECDF to deviate from the theoretical distribution. That said, the deviations would only affect the classification measures and the determination of the cutoff o^* whether optimally or not, but they would not affect the read reassignment procedure and the initial purging that retains all predicted real molecules (i.e. when we set $o^* = 1$) since the marginal distribution is not involved at this step.

Memory requirements. For data generated on the HiSeq 4000, the analysis workflow does not require computational requirements beyond what is found on a regular desktop with 32G of RAM. However, for NovaSeq 6000 data with a large number of multiplexed samples (e.g. $S > 8$), more memory would be needed - up to 150G if not more. In particular, the first step in the workflow consisting of joining data from all samples into a datatable keyed by cell-barcode, UMI, and gene ID is the most memory intensive.

Incorporating the sample barcode indices in the model. In this work, we formulated a model for index hopping that treats all the four distinct sample barcodes present in a given sample as identical. Even though we showed that the sample barcode index hopping rate can be derived from the model 3.2, we have not used information from the sample barcode indices to reassign reads or to purge phantom molecules. The model can potentially be expanded to incorporate the 4 sample barcodes, but such an extension would be accomplished at the cost of increased, if not prohibitive, computational and memory requirements since the workflow would need to start with the FASTQ files, not the output of the CellRanger pipeline and furthermore, the read count data table would need to be joined and keyed across 4 times the number of samples, potentially requiring more memory than would be available on some clusters. We would like to point out that the main reason we chose to formulate a model for index hopping at the level of samples and not individual sample barcodes is that in the end, sample index hopping between reads belonging to the same sample would not have any effect on downstream analyses since no phantom molecules would be generated when a read in a given sample swaps its sample barcode index with one of the other three barcode indices that are assigned to the same sample.

Supplementary Figures

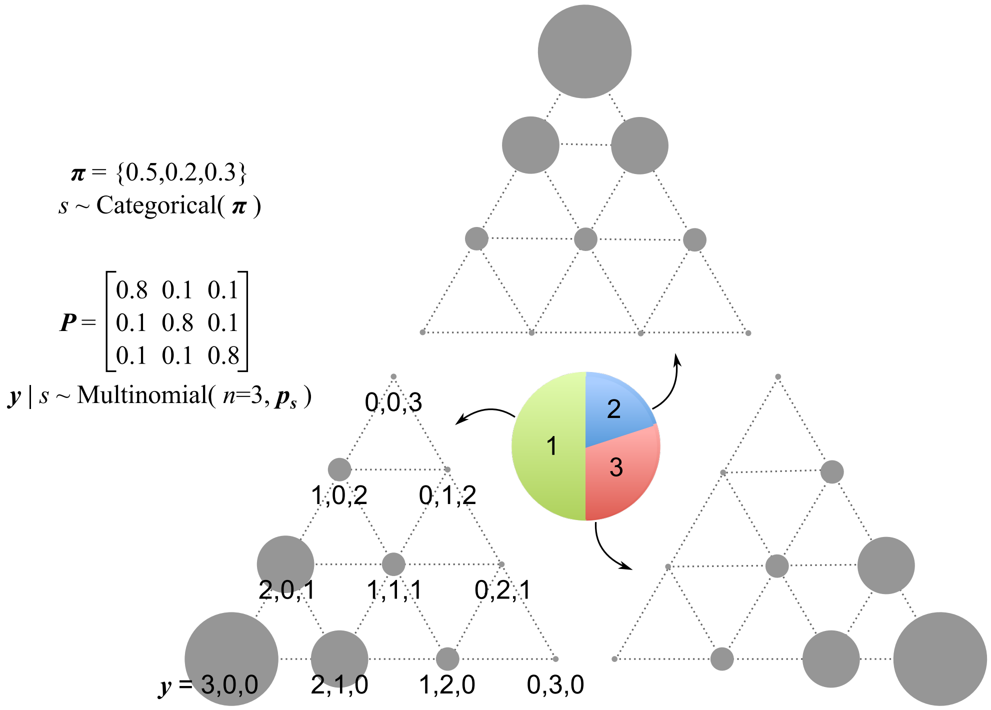

Supplementary Figure 1: **Schematic representation of the generative probabilistic model.** This toy example shows a molecule with three reads (PCR duplication level $r = 3$), in a multiplexed dataset of three samples. A molecule is drawn from samples 1 to 3 with a 0.5:0.2:0.3 probability (categorical distribution, represented with the pie chart in the middle). Conditional on this true sample, the apparent sample of the three reads follows a multinomial distribution (represented by the simplexes; the circle sizes are proportional to the conditional probabilities). The index hopping probability in this toy example is 0.2.

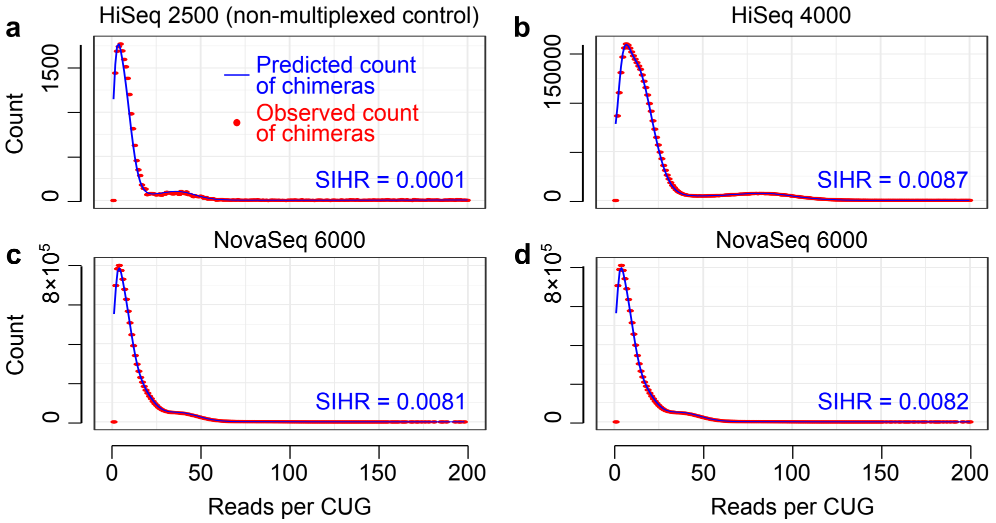

Supplementary Figure 2: Concordance between model fit and empirically observed chimeric counts. Each panel corresponds to a different previously published dataset. Each plot shows the fit on the absolute count of chimeras (for proportions, see **Figure 2**). (**a**) Mouse epithelial non-multiplexed HiSeq 2500 dataset [7]. (**b**) Mouse epithelial multiplexed HiSeq 4000 dataset [6]. (**c**) Tabula Muris NovaSeq 6000 dataset (Lane 1) [8]. (**d**) Tabula Muris NovaSeq 6000 dataset (Lane 2) [8].

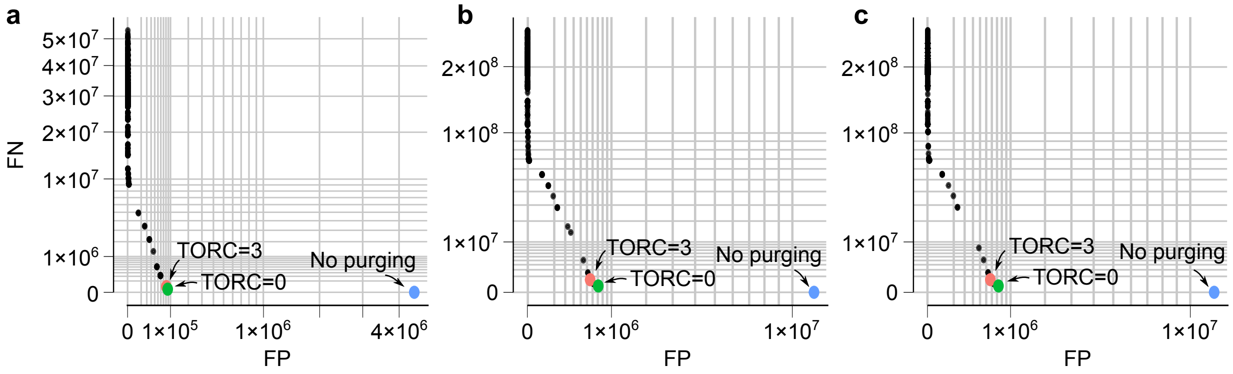

Supplementary Figure 3: The trade-off between false negatives (FN) and false positives(FP). The points correspond to the estimated number of FNs vs. FPs at different posterior probability cutoffs. (**a**) Mouse epithelial multiplexed HiSeq 4000 dataset [6]. (**b**) Tabula Muris NovaSeq 6000 dataset (Lane 1) [8]. (**c**) Tabula Muris NovaSeq 6000 dataset (Lane 2) [8]. In each panel, the FP/FN values when the data are kept as it is (no purging), or reads are reassigned without further filtering by posterior probability (TOR cutoff=0) are also shown.

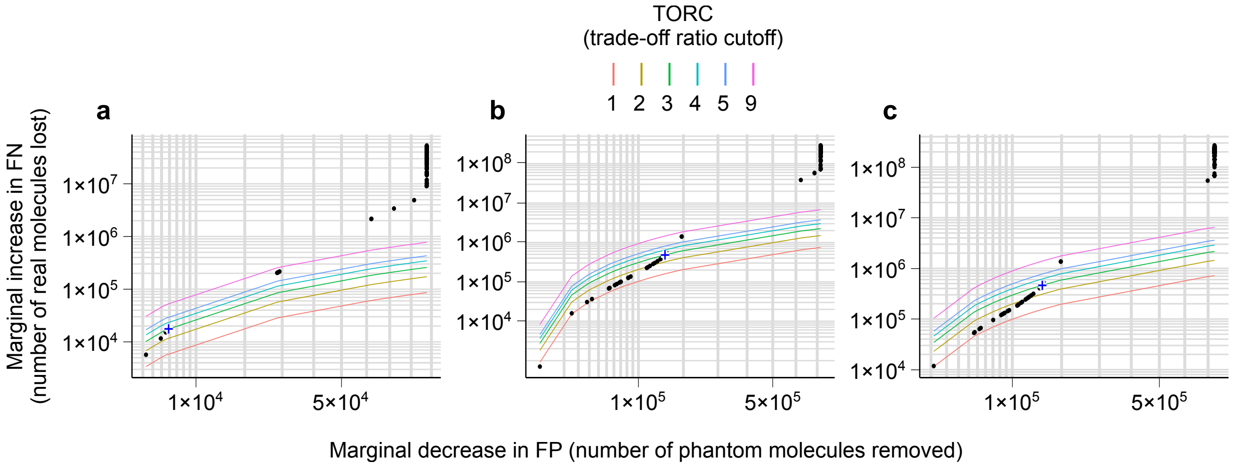

Supplementary Figure 4: Marginal increase in false negatives against the marginal decrease in false positives. Dots correspond to estimated marginal decrease/increase of false positives/negatives at different posterior probability cutoffs for each dataset. The colored lines show the decision curves for different trade-off ratio (TOR) cutoff values. At any given TOR cutoff value, the point that is on or immediately below the corresponding decision curve will be selected as the posterior probability cutoff. (**a**) Mouse epithelial multiplexed HiSeq 4000 dataset [6]. (**b**) Tabula Muris NovaSeq 6000 dataset (Lane 1) [8]. (**c**) Tabula Muris NovaSeq 6000 dataset (Lane 2) [8].

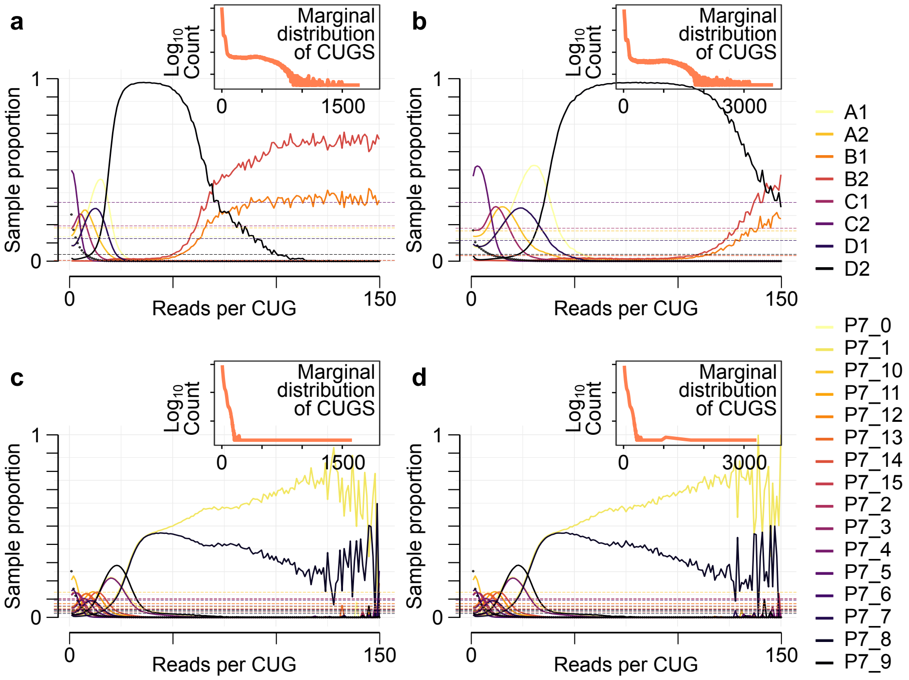

Supplementary Figure 5: The molecular proportions complexity (MPC) profile plots for four previously published datasets. Each curve represents the proportion of library complexity (i.e. fraction of molecules) at the indicated PCR amplification level r that originate from a given sample. The collection of the curves in each panel make up the MPC profile of the dataset. The horizontal dotted lines represent the marginal library complexity proportions. Note that while the plots show r up to a maximum of 150 due to sparsity of observations beyond this range, the corresponding subplots in the upper right corner of each panel shows the distribution of the number of observations for the entire observed range of r.

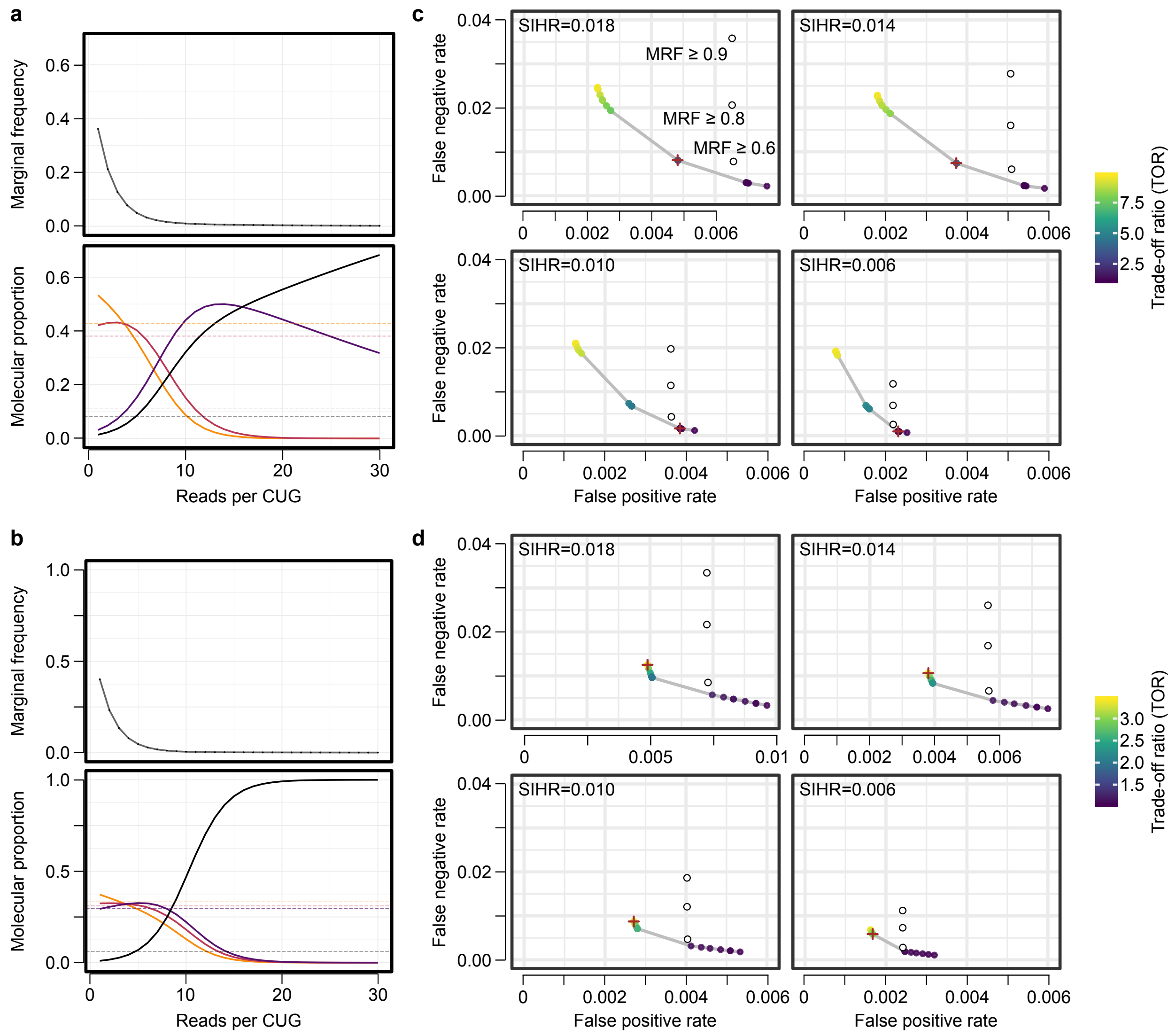

Supplementary Figure 6: **Evaluation of model-based purging of phantom molecules using simulated data**. (**a-b**) Two simulated molecular proportion complexity (MPC) profiles that were used as the basis for simulating datasets. In each panel, the top graph shows the marginal distribution of CUGs, whereas the bottom panel shows the molecular proportions of each of the four simulated samples, conditional on the PCR amplification level (i.e. conditional on reads per CUG). (**c-d**) Performance on simulated data. The colored dots represent FP and FN proportions for distinguishing true molecules from phantom molecules, after filtering at different posterior probability cutoffs. The color of each dot represents the trade-off ratio, i.e. the additional number of real molecules lost for every phantom molecule that is correctly purged. The point that corresponds to a TOR cutoff of 3 is marked with a plus. For each MPC profile, the simulations were performed for four evenly spaced values of SIHR (sample index hopping rate). The open circles show the FN/FP values obtained by the heuristic approach [6] with three choices of the minimum read fraction (MRF) threshold – from top to bottom: 0.9, 0.8, and 0.6. (**c**) Performance on data simulated based on the MPC profile shown in (a). (**d**) Performance on data simulated based on the MPC profile shown in (b).

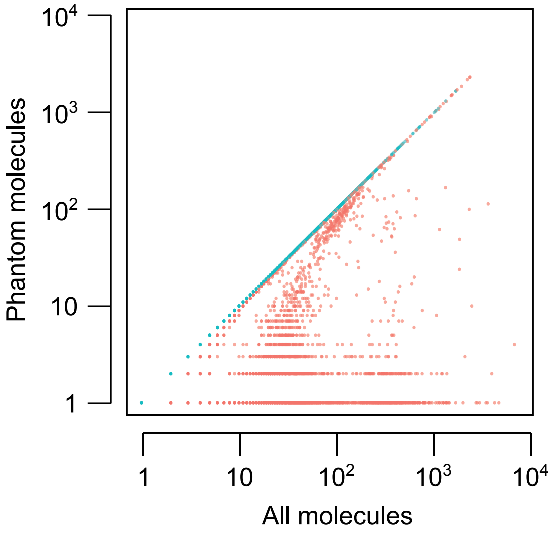

Supplementary Figure 7: **The ratio of phantom to total molecules per cell**. Each dot represents one cell that is affected by index hopping. Data correspond to sample S2 from the multiplexed validation dataset (i.e. two libraries with known ground truth multiplexed on the same HiSeq 4000 lane). Blue points are cells whose molecules are all phantom. the percentage of cells that contain at least one phantom molecule is approximately 20%.

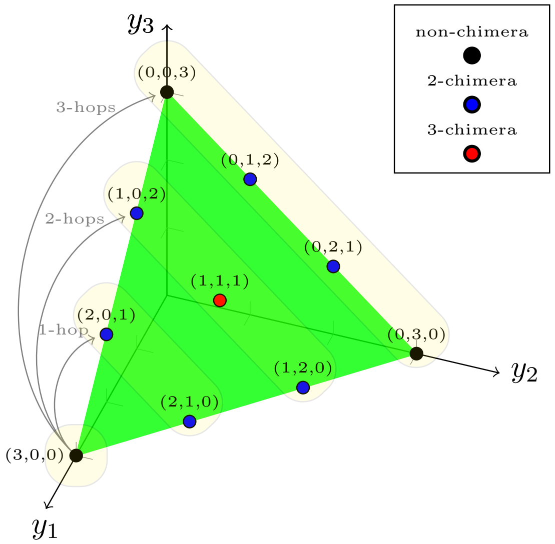

Supplementary Figure 8: **The 3rd component of Pascal's 3-Simplex denoted by** $\wedge_3^3$. All 10 possible outcomes (at read count sum r=3) for three samples (S=3) are arranged on the simplex. The sample of origin is $s = 1$ as shown by the arrows. Outcomes can be classified according to the number of observed molecules (i.e. k-chimera) or the number of hopped reads. The four outcomes corresponding to all 3 reads hopping over are called *fugues*. The two fugue outcomes in the vertices are termed *non-chimeric fugues* whereas the other two on the edge are termed *chimeric fugues*

Supplementary Tables

Supplementary Table 1: **Estimates of the sample index hopping rate for one non-multiplexed and three multiplexed sample libraries.** $\hat{p}$ is the estimate of the model parameter (i.e. the complement of the sample index hopping rate, SIHR); SBIHR is the sample barcode index hopping rate (see **Methods** 3.2). $m.e.$ is the margin of error corresponding to a 99% confidence interval; u is the molecule inflation factor, a summary measure that represents a lower limit on the number of phantom molecules in the data; g is the proportion of fugue observations, i.e. CUGs whose reads are all affected by index hopping (and therefore none of their reads represent a real molecule); and PPM=$(u+g)/(1+u+g)$ is the Proportion of Phantom Molecules in the dataset. The HiSeq 2500 dataset (control) represents eight mouse epithelial sample libraries that were sequenced separately (non-multiplexed) on different HiSeq 2500 lanes [7]. The HiSeq4000 dataset corresponds to eight mouse epithelial sample libraries that were multiplexed on a single HiSeq 4000 lane [6]. The NovaSeq L1 and NovaSeq L2 datasets correspond to the same set of 16 sample libraries that were multiplexed on two NovaSeq 6000 lanes [8].

dataset	$\hat{p}$	**SIHR**	**SBIHR**	$m.e.$	u	g	PPM
HiSeq 2500	0.99990	0.00010	0.00011	$\pm 1.11 \times 10^{-6}$	0.00060	0.00003	0.00062
HiSeq 4000	0.99130	0.00866	0.00960	$\pm 9.96 \times 10^{-6}$	0.08169	0.00147	0.07688
NovaSeq L1	0.99188	0.00812	0.00853	$\pm 4.73 \times 10^{-6}$	0.04131	0.00206	0.04167
NovaSeq L2	0.99177	0.00823	0.00864	$\pm 5.25 \times 10^{-6}$	0.04187	0.00210	0.04222

Supplementary Table 2: Estimating false positive (FP) and false negative (FN) rates after purging molecules at a given posterior probability cutoff. The table shows the confusion matrix for possible outcomes for classifying molecules as real or phantom. Given a data table of read counts with L observations, the total number of molecules would be $(1 + u) \times L$ where u is the *molecule inflation factor*. The variable o^* denotes the proportion of observations that fall above posterior probability threshold q^* such that $o^* \times L$ would be the number of molecules we label as real. The constant g is the proportion of fugue observations estimated for that given dataset. See **Supplementary Methods** for how u, g, and FP(qr^*) are calculated.

Truth \ Prediction	**Phantom**	**Real**	**Total**
**Phantom**	TN $=u + g-$FP	FP(qr^*)	u $+g$
**Real**	FN =FP $+1 - o^* - g$	TP $= o^*$ - FP	1-g
**Total**	u+1-o^*	o^*	1+u

Supplementary Table 3: Classification performance comparison of four different approaches. Results correspond to a multiplexed dataset of two libraries in which the ground truth is known (i.e. the source sample of each molecule is determined by sequencing the same two libraries on two separate lanes). *No purging* corresponds to the multiplexed data as is. *No discarding* (Purging at TOR=0) corresponds to reassignment of reads to the sample with the largest posterior probability, but without further filtering. *Discarding* (Purging at TOR=3) represents the results after discarding the molecules that have a posterior probability lower than a specified cutoff, with the cutoff determined so as to achieve a trade-off ratio of 3 (see Methods). *Min fraction* corresponds to a previous heuristic approach [6] based on keeping the molecules for which at least 80% of reads are assigned to the same sample. The number of fugue molecules in the dataset is 9995 (theoretical FP minimum). FP: false positive count (phantom molecules that are mistakenly classified as true molecules); FN: false negative count (real molecules that are mistakenly classified as phantom molecules); TP: true positive count (real molecules that are classified as real); TN: true negative count (phantom molecules that are classified as phantom); FPR: false positive rate = FP/(FP+TN); FNR: false negative rate = FN/(FN+TP).

Approach	FP	FN	TP	TN	FPR	FNR
No purging	485399	0	9242100	0	1.0000	0.0000
Purging at TOR=0	12585	2590	9239510	472814	0.0259	0.0003
Purging at TOR=3	10116	7685	9234416	475283	0.0208	0.0008
Min read fraction ≥ 0.8	10000	16094	9226006	475399	0.0206	0.0017

Supplementary Table 4: Performance on simulated data. The table shows the false positive and false negative proportions for the default cutoff value of the minimum read fraction (MRF) method [6] and our probabilistic modeling approach for four evenly spaced values of p (the complement of the sample index hopping rate). We used a trade-off ratio (TOR) cutoff of 3 for our method. Note that the TOR after purging corresponds to the largest TOR value obtainable that is still smaller than TOR cutoff of 3.

p	Cutoff	FP	FN
0.982	MRF$\geq$ 0.80	0.00374	0.02210
0.982	TOR=2.35	0.00343	0.00492
0.986	MRF$\geq$ 0.80	0.00291	0.01696
0.986	TOR=2.43	0.00306	0.00230
0.990	MRF$\geq$ 0.80	0.00208	0.01195
0.990	TOR=2.98	0.00212	0.00205
0.994	MRF$\geq$ 0.80	0.00124	0.00707
0.994	TOR=2.98	0.00127	0.00122

Supplementary Table 5: Extent of contamination by sample for mouse epithelial non-multiplexed HiSeq 2500 dataset (control) and a multiplexed HiSeq 4000 dataset. The column *Molecules* lists the number of total molecules in each sample. The *Breakdown* columns list the proportion of predicted real molecules (*Real*), molecules discarded at TOR cutoff=0 (*Phantom*^{0}), and additional molecules discarded when TOR cutoff is increased to 3 (*Phantom*^{3}). The *Proportions* columns list the marginal proportions of molecules and reads across the entire dataset. RMR: the ratio of reads to molecules. Note the small fraction of molecules discarded in the non-multiplexed dataset, which serves as a negative control that should not be affected by our purging approach.

		**Breakdown**			**Proportions**		
Sample	Molecules	Real	Phantom^0	Phantom^3	Molecules	Reads	RMR
**HiSeq 2500 Dataset**							
A1	5.94×10^6	0.9986	0.0014	0.0000	0.13	0.13	6.1
A2	8.02×10^6	0.9997	0.0003	0.0000	0.18	0.13	4.5
B1	0.15×10^6	0.9795	0.0205	0.0000	0.00	0.13	228.8
B2	0.20×10^6	0.9865	0.0135	0.0000	0.00	0.13	177.8
C1	8.51×10^6	0.9997	0.0003	0.0000	0.19	0.12	3.7
C2	14.19×10^6	0.9998	0.0002	0.0000	0.32	0.12	2.3
D1	5.45×10^6	0.9995	0.0004	0.0001	0.12	0.11	5.5
D2	1.63×10^6	0.9984	0.0016	0.0000	0.04	0.12	20.1
**Hiseq 4000 Dataset**							
A1	7.03×10^6	0.9662	0.0337	0.0001	0.12	0.13	11.6
A2	9.59×10^6	0.9776	0.0222	0.0002	0.17	0.13	8.6
B1	1.82×10^6	0.1351	0.8643	0.0006	0.03	0.13	44.1
B2	1.75×10^6	0.1754	0.8239	0.0006	0.03	0.14	50.1
C1	10.46×10^6	0.9781	0.0215	0.0004	0.18	0.12	7.3
C2	18.65×10^6	0.9874	0.0120	0.0006	0.32	0.12	4.0
D1	6.56×10^6	0.9684	0.0314	0.0002	0.11	0.11	10.2
D2	2.16×10^6	0.8787	0.1207	0.0006	0.04	0.12	35.1

Supplementary Table 6: **Extent of contamination by sample for the Tabula Muris NovaSeq 6000 datasets**. The column *Molecules* lists the number of total molecules in each sample. The *Breakdown* columns list the proportion of predicted real molecules (*Real*), molecules discarded at TOR cutoff=0 ($Phantom^0$), and additional molecules discarded when TOR cutoff is increased to 3 ($Phantom^3$). The *Proportions* columns list the marginal proportions of molecules and reads across the entire dataset. RMR: the ratio of reads to molecules.

		**Breakdown**			**Proportions**		
Sample	Molecules	Real	$Phantom^0$	$Phantom^3$	Molecules	Reads	RMR
**NovaSeq 6000 Lane 1 Dataset**							
P7_0	13.54×10^6	0.9316	0.0672	0.0012	0.05	0.07	7.9
P7_1	8.16×10^6	0.8653	0.1343	0.0004	0.03	0.08	14.5
P7_2	28.02×10^6	0.9754	0.0214	0.0032	0.10	0.06	2.9
P7_3	28.58×10^6	0.9760	0.0214	0.0026	0.10	0.06	3.1
P7_4	8.48×10^6	0.9101	0.0898	0.0001	0.03	0.06	10.1
P7_5	17.40×10^6	0.9678	0.0302	0.0001	0.06	0.05	4.5
P7_6	26.17×10^6	0.9773	0.0181	0.0045	0.09	0.04	2.3
P7_7	11.69×10^6	0.9512	0.0478	0.0010	0.04	0.05	5.7
P7_8	5.99×10^6	0.8247	0.1753	0.0000	0.02	0.07	17.3
P7_9	10.49×10^6	0.9146	0.0849	0.0005	0.04	0.07	10.1
P7_10	38.68×10^6	0.9837	0.0132	0.0031	0.14	0.06	2.4
P7_11	16.20×10^6	0.9549	0.0437	0.0014	0.06	0.07	6.1
P7_12	21.28×10^6	0.9663	0.0315	0.0022	0.08	0.07	4.8
P7_13	20.80×10^6	0.9681	0.0298	0.0021	0.07	0.07	4.9
P7_14	12.92×10^6	0.9470	0.0523	0.0007	0.05	0.06	6.8
P7_15	12.81×10^6	0.9600	0.0386	0.0014	0.05	0.05	5.4
**NovaSeq 6000 Lane 2 Dataset**							
P7_0	13.59×10^6	0.9309	0.0012	0.0679	0.05	0.07	7.9
P7_1	8.20×10^6	0.8645	0.1351	0.0004	0.03	0.08	14.4
P7_2	28.11×10^6	0.9750	0.0218	0.0032	0.10	0.06	2.9
P7_3	28.67×10^6	0.9756	0.0217	0.0026	0.10	0.06	3.1
P7_4	8.51×10^6	0.9088	0.0910	0.0001	0.03	0.06	10.1
P7_5	17.43×10^6	0.9674	0.0306	0.0020	0.06	0.05	4.5
P7_6	26.25×10^6	0.9770	0.0184	0.0046	0.09	0.04	2.3
P7_7	11.72×10^6	0.9506	0.0485	0.0010	0.04	0.05	5.7
P7_8	6.03×10^6	0.8238	0.1762	0.0000	0.02	0.07	17.3
P7_9	10.53×10^6	0.9139	0.0856	0.0005	0.04	0.07	10.1
P7_10	38.78×10^6	0.9834	0.0135	0.0031	0.14	0.06	2.4
P7_11	16.25×10^6	0.9543	0.0443	0.0014	0.06	0.07	6.1
P7_12	21.34×10^6	0.9657	0.0320	0.0022	0.08	0.07	4.8
P7_13	20.85×10^6	0.9676	0.0303	0.0022	0.07	0.07	4.9
P7_14	12.96×10^6	0.9463	0.0530	0.0007	0.05	0.06	6.8
P7_15	12.84×10^6	0.9595	0.0392	0.0014	0.05	0.05	5.4

Supplementary Table 7: **Effect of contamination on cell calling in mouse epithelial non-multiplexed HiSeq 2500 (control) and multiplexed HiSeq 4000 datasets**. The *cell* and *empty* columns list the number of cell-barcodes that were categorized as RNA-containing cells or background cells (empty droplets), respectively. The *Phantom* column enumerates the cells and empty droplets that disappear once phantom molecules are purged; The *Consensus* column enumerates the cells and empty droplets that maintain the same status no matter whether the phantom molecules were purged or not; The *Reclassified* column represents the number of cell-barcodes that were re-classified (transitioned) as empty droplet or cell after purging the phantom molecules; The *Called Cells* columns shows the number of cell-barcodes that were categorized as RNA-containing cells before and after purging. Note the small number of changes in the non-multiplexed dataset, which serves as a negative control that should not be affected by our purging approach.

	**Phantom**		**Consensus**		**Reclassified**		**Called Cells**	
Sample	Cell	Empty	Cell	Empty	Cell	Empty	Before	After
**HiSeq 2500 Dataset**								
A1	0	1,332	409	123,769	1	0	409	410
A2	0	770	564	138,948	0	2	566	564
B1	4	1,258	199	26,138	0	17	220	199
B2	0	1,136	16	29,971	0	0	16	16
C1	0	808	739	145,283	0	0	739	739
C2	0	668	1,316	195,614	4	1	1,317	1,320
D1	0	778	685	98,969	1	0	685	686
D2	0	975	160	61,353	1	0	160	161
**HiSeq 4000 Dataset**								
A1	0	12,478	401	138,117	13	105	506	414
A2	0	11,188	565	156,913	13	105	670	578
B1	0	70,193	16	40,016	0	1,007	1,023	16
B2	0	65,001	20	45,503	0	885	1,005	20
C1	0	11,729	730	168,503	0	118	1,474	1,323
C2	0	10,087	1,318	232,404	5	154	725	676
D1	0	11,609	661	119,473	15	64	725	676
D2	0	15,912	165	82,287	7	29	194	172

Supplementary Table 8: **Effect of contamination on cell calling in the Tabula Muris NovaSeq 6000 datasets**. The *cell* and *empty* columns list the number of cell-barcodes that were categorized as RNA-containing cells or background cells (empty droplets), respectively. The *Phantom* column enumerates the cells and empty droplets that disappear once phantom molecules are purged; The *Consensus* column enumerates the cells and empty droplets that maintain the same status no matter whether the phantom molecules were purged or not; The *Reclassified* column represents the number of cell-barcodes that were re-classified (transitioned) as empty droplet or cell after purging the phantom molecules; The *Called Cells* columns shows the number of cell-barcodes that were categorized as RNA-containing cells before and after purging.

	**Phantom**		**Consensus**		**Reclassified**		**Called Cells**	
Sample	Cell	Empty	Cell	Empty	cell	empty	Before	After
			**NovaSeq 6000 Lane 1 Dataset**					
P7_0	0	61,772	1,119	245,922	156	196	1,315	1,275
P7_1	0	73,677	551	231,820	105	223	774	656
P7_2	0	53,833	4,037	306,452	13	21	4,058	4,050
P7_3	0	49,661	3,974	330,196	26	26	4,000	4,000
P7_4	0	63,917	927	250,569	29	69	996	956
P7_5	0	47,393	1,969	316,514	37	12	1,981	2,006
P7_6	0	45,701	7,268	304,202	63	17	7,285	7,331
P7_7	0	63,518	733	210,349	21	11	744	754
P7_8	0	95,414	1,089	175,093	21	108	1,197	1,110
P7_9	0	76,342	2,306	238,232	8	89	2,395	2,314
P7_10	0	41,213	2,449	375,818	19	11	2,460	2,468
P7_11	0	64,228	3,474	259,465	1	32	3,506	3,475
P7_12	0	59,249	3,027	291,810	16	28	3,055	3,043
P7_13	0	53,450	3,383	305,486	10	26	3,409	3,393
P7_14	0	64,676	2,601	244,136	12	28	2,629	2,613
P7_15	0	54,118	2,753	242,666	12	8	2,761	2,765
			**NovaSeq 6000 Lane 2 Dataset**					
P7_0	0	62,320	947	246,383	358	213	1,160	1,305
P7_1	0	73,684	611	232,303	43	224	835	654
P7_2	0	53,673	4,036	307,442	11	22	4,058	4,047
P7_3	0	50,035	3,981	331,060	24	25	4,006	4,005
P7_4	0	64,176	826	251,665	17	173	999	843
P7_5	0	47,130	2,023	317,367	40	18	2,041	2,063
P7_6	0	46,122	7,264	305,496	76	16	7,280	7,340
P7_7	0	63,731	732	211,401	23	11	743	755
P7_8	0	95,323	1,089	176,709	24	120	1,205	1,113
P7_9	0	76,811	2,305	239,238	13	95	2,400	2,318
P7_10	0	41,633	2,451	376,946	21	13	2,464	2,472
P7_11	0	64,495	3,476	261,000	0	38	3,514	3,476
P7_12	0	59,618	3,029	293,207	16	33	3,062	3,045
P7_13	0	54,118	3,389	306,711	7	27	3,416	3,396
P7_14	0	65,386	2,597	245,290	15	33	2,630	2,612
P7_15	0	54,121	2,757	244,201	6	8	2,765	2,763

Supplementary Table 9: **Toy dataset of sequencing reads**. This table depicts three multiplexed samples (i.e. $S = 3$) in which each observation is a sequencing read with label m (i.e. the combination of c, u, and g, which denote the cell-barcode, UMI, and gene, respectively), a true source sample s, and an observed sample d_i, $i = 1, 2, 3$. In a typical experiment, a single lane can generate anywhere from 350 million to 2.5 billion sequencing reads.

label	l	s	d_1	d_2	d_3
$c_1u_2g_1$	1	1	0	1	0
$c_1u_1g_1$	2	2	1	0	0
$c_1u_3g_2$	3	2	1	0	0
$c_1u_3g_2$	3	2	1	0	0
$c_1u_3g_2$	3	2	0	1	0
$c_2u_1g_1$	4	1	0	1	0
$c_2u_1g_1$	4	1	0	0	1
$c_2u_1g_1$	4	1	0	0	1
$\vdots$	$\vdots$	$\vdots$	$\vdots$	$\vdots$	$\vdots$

Supplementary Table 10: **Toy dataset of read counts and corresponding deduplicated counts (i.e. molecules)**. The data table shows 4 out of the 10 possible outcomes at read count sum level $r = 3$ for multiplexed data with $S = 3$ samples (see **Supplementary Figure 8**). Each of the four observations are associated with a unique *cell-umi-gene* label l and an unobservable true sample of origin $s = 1$. For each read count $\boldsymbol{y}$, a vector of deduplicated read counts or molecules $\boldsymbol{x}$ is given. The chimeric number k denotes the number of molecules observed in each outcome and f denotes the number of phantom molecules. Although the chimeric vs. non-chimeric state of an observation can be directly seen from the data, the fugue vs. non-fugue state of an observation cannot be directly inferred, since it depends on knowing the true origin of the reads (i.e. the latent variable s). When there is no index hopping, the only outcomes possible are those classified as non-chimeric non-fugues.

					$\boldsymbol{y}$ (reads)			$\boldsymbol{x}$ (molecules)			
l	r	k	f	s	y_1	y_2	y_3	x_1	x_2	x_3	category
1	3	1	0	1	3	0	0	1	0	0	non-chimeric non-fugue
2	3	1	1	1	0	3	0	0	1	0	non-chimeric fugue
3	3	2	1	1	1	2	0	1	1	0	chimeric non-fugue
4	3	2	2	1	0	1	2	0	1	1	chimeric fugue
⋮	⋮	⋮	⋮	⋮	⋮	⋮	⋮	⋮	⋮	⋮	⋮

Supplementary References

[1] MacConaill, L. E. *et al.* Unique, dual-indexed sequencing adapters with UMIs effectively eliminate index cross-talk and significantly improve sensitivity of massively parallel sequencing. *BMC Genomics* **19**, 30 (2018).

[2] Illumina. Effects of index misassignment on multiplexing and downstream analysis. White paper at https://www.illumina.com/content/dam/illumina-marketing/documents/products/whitepapers/index-hopping-white-paper-770-2017-004.pdf (2017). Accessed: 2020-04-16.

[3] Hadfield, J. Index mis-assignment between samples on HiSeq 4000 and X-Ten. http://enseqlopedia.com/2016/12/index-mis-assignment-between-samples-on-hiseq-4000-and-x-ten/ (2016). Accessed: 2020-04-16.

[4] Sinha, R. *et al.* Index switching causes "spreading-of-signal" among multiplexed samples in Illumina HiSeq 4000 DNA sequencing. Preprint at https://www.biorxiv.org/content/10.1101/125724v1 (2017).

[5] Griffiths, J. A., Richard, A. C., Bach, K., Lun, A. T. & Marioni, J. C. Detection and removal of barcode swapping in single-cell RNA-seq data. *Nat Commun* **9**, 2667 (2018).

[6] Vodák, D. *et al.* Sample-Index Misassignment Impacts Tumour Exome Sequencing. *Sci Rep* **8**, 5307 (2018).

[7] Yao, Y. *et al.* Exploiting antigen receptor information to quantify index switching in single-cell transcriptome sequencing experiments. *PLOS ONE* **13**, 1-17 (2018).

[8] Costello, M. *et al.* Characterization and remediation of sample index swaps by non-redundant dual indexing on massively parallel sequencing platforms. *BMC Genomics* **19**, 332 (2018).

[9] Larsson, A. J., Stanley, G., Sinha, R., Weissman, I. L. & Sandberg, R. Computational correction of index switching in multiplexed sequencing libraries. *Nat Methods* **15**, 305–307 (2018).

[10] Zerbino, D. R. *et al.* Ensembl 2018. *Nucleic Acids Research* **46**, D754–D761 (2018).

[11] Youden, W. J. Index for rating diagnostic tests. *Cancer* **3**, 32-35 (1950).

[12] Lun, A. T. L. *et al.* EmptyDrops: distinguishing cells from empty droplets in droplet-based single-cell RNA sequencing data. *Genome Biology* **20**, 63 (2019).

[13] Bach, K. *et al.* Differentiation dynamics of mammary epithelial cells revealed by single-cell RNA sequencing. *Nat Commun* **8**, 2128 (2017).

[14] The Tabula Muris Consortium. Single-cell transcriptomics of 20 mouse organs creates a Tabula Muris. *Nature* **562**, 367–372 (2018).
